# Variability of Amyloid Propensity in Imperfect Repeats of CsgA Protein of *Salmonella enterica* and *Escherichia coli*

**DOI:** 10.3390/ijms22105127

**Published:** 2021-05-12

**Authors:** Natalia Szulc, Marlena Gąsior-Głogowska, Jakub W. Wojciechowski, Monika Szefczyk, Andrzej M. Żak, Michał Burdukiewicz, Malgorzata Kotulska

**Affiliations:** 1 Department of Biomedical Engineering, Faculty of Fundamental Problems of Technology, Wroclaw University of Science and Technology, Wybrzeże Wyspiańskiego 27, 50-370 Wrocław, Poland; natalia.szulc@pwr.edu.pl (N.S.); marlena.gasior-glogowska@pwr.edu.pl (M.G.-G.); jakub.wojciechowski@pwr.edu.pl (J.W.W.); 2 LPCT, CNRS, Université de Lorraine, F-54000 Nancy, France; 3 Department of Bioorganic Chemistry, Faculty of Chemistry, Wroclaw University of Science and Technology, Wybrzeże Wyspiańskiego 27, 50-370 Wrocław, Poland; monika.szefczyk@pwr.edu.pl; 4 Electron Microscopy Laboratory, Faculty of Mechanical Engineering, Wroclaw University of Science and Technology, Wybrzeże Wyspiańskiego 27, 50-370 Wrocław, Poland; andrzej.zak@pwr.edu.pl; 5 Clinical Research Centre, Medical University of Białystok, Jana Kilińskiego 1, 15-089 Białystok, Poland; 6 Institute of Biochemistry and Biophysics, Polish Academy Sciences, 02-106 Warsaw, Poland; 7 Faculty of Natural Sciences, Brandenburg University of Technology Cottbus-Senftenberg, 01968 Senftenberg, Germany

**Keywords:** functional amyloids, curli, aggregation, biofilm, ATR-FTIR, FT-Raman

## Abstract

CsgA is an aggregating protein from bacterial biofilms, representing a class of functional amyloids. Its amyloid propensity is defined by five fragments (R1–R5) of the sequence, representing non-perfect repeats. Gate-keeper amino acid residues, specific to each fragment, define the fragment’s propensity for self-aggregation and aggregating characteristics of the whole protein. We study the self-aggregation and secondary structures of the repeat fragments of *Salmonella enterica* and *Escherichia coli* and comparatively analyze their potential effects on these proteins in a bacterial biofilm. Using bioinformatics predictors, ATR-FTIR and FT-Raman spectroscopy techniques, circular dichroism, and transmission electron microscopy, we confirmed self-aggregation of R1, R3, R5 fragments, as previously reported for *Escherichia coli*, however, with different temporal characteristics for each species. We also observed aggregation propensities of R4 fragment of *Salmonella enterica* that is different than that of *Escherichia coli*. Our studies showed that amyloid structures of CsgA repeats are more easily formed and more durable in *Salmonella enterica* than those in *Escherichia coli*.

## 1. Introduction

Functional amyloids are spread across nearly the whole tree of life, including archaea, bacteria, fungi, protozoa, and viruses [1]. Although functional amyloids, similarly to pathological amyloids, self-assemble into fibers, their aggregates are involved in a wide range of crucial molecular tasks, including hormone storage, signaling, enhancing cell adhesion, and biofilm formation [2]. Aside from these functionalities, some bacterial functional amyloids constitute a proteinaceous skeleton of the extracellular matrix, called biofilm. Bacteria produce biofilms to create an environment protecting them from adverse conditions. This ability is widespread in nature and can be seen as one of the most common survival strategies adopted by bacteria [3]. It is estimated that between 40% and 80% of all bacterial cells are part of biofilms [4].

As the ability to form stable biofilms depends on the bacterial natural niche and their genotypic characteristics [5], it is also affected by the phylogenetic variability of functional amyloids involved in this process. Here, we focus on one of the best-studied amyloids involved in biofilm formation, curli fibers [6]. Curli form non-branched fibrils on the cell surface, which are very resistant to degradation by proteases and detergents. A primary structural component of these fibrils is CsgA protein. The most widely studied functional amyloid is CsgA of *Escherichia coli* (*E. coli*). It is a 151 amino acid long protein, including N terminal signaling peptide, which is proteolytically cleaved, and a core amyloid domain transported outside the cell by CsgG protein [7]. CsgA forms amyloid fibrils along with CsgB protein, which enhances the fibril formation [8]. The curli homologs are prevalent among other Enterobacteriaceae, although in many cases they exhibit a large structural diversity. The CsgA sequence consists of five imperfect repeats labeled as R1–R5 fragments, which, in *E. coli,* follows a common pattern S-[X]5-Q-[X]-G-[X]-G-N-[X]-A-[X]3-Q. The motif depends on bacterial species and it can be altered in curli of other species. Fragments R1 and R5, as the most amyloidogenic, are critical for seeding and the curli formation in *E. coli* [9,10]. The other three fragments are less prone to aggregation or non-aggregating at all.

Despite their variability, it has been shown that even very distant CsgA homologs can together contribute to the formation of the heterogeneous curli fibrils [11]. This phenomenon probably occurs due to the widespread presence of imperfect repeats, which appear more or less commonly in all CsgA and CsgA-like proteins [12].

This ability to interact with other amyloids is also a source of putative negative effect of curli. It has been shown that CsgA produced by the gut microflora can promote aggregation of human proteins by the phenomenon called cross-seeding, for example, facilitating aggregation of α-synuclein or amyloid Aβ, which are involved in human amyloid diseases [13,14]. This poorly understood phenomenon, termed “mapranosis” (microbiota-associated proteopathy and neuroinflammation), may lead to contribution of the microbiome to neurodegenerative diseases [15].

Currently, one of the challenges in understanding the self-assembly propensity of CsgA is the evolutionary variability of imperfect repeats. To shed more light on the structural determinants of the amyloid propensity of imperfect repeats, we studied the behavior of CsgA homolog from *Salmonella enterica* (*S. enterica*), a common foodborne pathogen that creates many challenges in medicine and food industry [16]. Its CsgA has a slightly altered motif; one of the glycines is not always present (R1 lacking the first glycine, R3 and R4 lacking the second one). Although it is very similar to CsgA from *E. coli*, the aggregation kinetics of these two proteins may be different. Therefore, we compare in vitro the aggregation propensities of CsgA fragments *S. enterica* with *E. coli* strain K12. The knowledge of their properties may lead to better understanding of the principles of functional amyloid aggregation and, thus, help in developing anti-biofilm agents [17].

## 2. Results

### 2.1. Sequence Alignment

**Figure 1:**
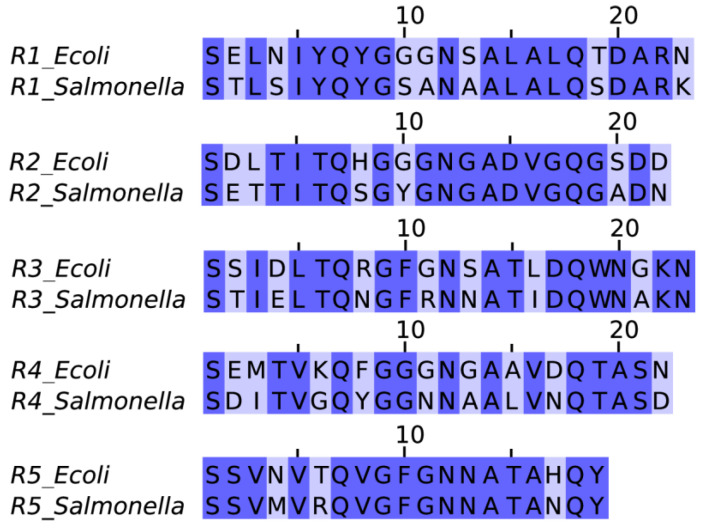
Pairwise sequence alignment between CsgA fragments of *E. coli* and *S. enterica* bacteria. The differences in the amino acid compositions are highlighted with light purple.

CsgA proteins from *E. coli* and *S. enterica* are closely related homologues with 75% of identity, as calculated by BLAST [18]. To further investigate differences between corresponding fragments, pairwise alignments were performed for each pair of the fragments (Figure 1). The alignment confirmed that the sequences were highly similar to their counterparts. Almost all of the peptides share similar features: they are rather hydrophilic, with their ends often containing charged amino acids and a highly flexible glycine rich linker in the middle. Such an architecture suggests the propensity to form beta arch structure, which agrees with the computational results of ArchCandy for almost all of these sequences [19] (Table S1 in SI).

However, their aggregation propensities may differ in terms of physicochemical properties due to point mutations of CsgA genes. For example, their hydrophobicity (as shown by GRAVY index score, Table S2 in SI) or pI can be changed. Mutations of aromatic amino acids, stabilizing amyloid peptide structures by forming π stacking, are of great importance to the aggregates [20]. Similarly, charged residues may play an important role in the amyloid propensity of CsgA proteins, depending on their location [21,22,23]. Not only the contributing residues affect the peptide aggregation susceptibility but also the sequence order is of great importance. This fact was discovered by statistical analyses, which led to the release of several bioinformatics predictors. The sequence alignment of corresponding pairs from both bacterial species shows that different amyloid propensities of the CsgA fragments could not be excluded.

### 2.2. Bioinformatics Analysis

We applied bioinformatics tools to analyze amyloidogenic propensity of CsgA fragments with imperfect repeats from *E. coli* and *S. enterica*. The objective was identification of possible differences in their amyloidogenic propensities, and potential impact on different aggregation of the whole proteins. The results directed us to further in vitro studies of the fragments.

The analysis was performed with our amyloid predictors, which showed a very high accuracy, AmyloGram [24] and PATH [25]. Both methods were trained on hexapeptides collected in AmyLoad [26] and Waltz 2.0 databases [27]. The only difference between corresponding fragments was obtained for R4. In this case, despite significant similarity of the sequences, AmyloGram provided different results. R4 fragment from *S. enterica* was reported as amyloidogenic, while R4 from *E. coli* was reported as non-amyloidogenic. Therefore, this fragment was selected as a candidate that may have different aggregation propensities. For a comparison, we also applied several other bioinformatic predictors, such as Pasta 2.0 [28], Waltz [29], AmylPred2 [30], FoldAmyloid [31], MetAmyl [32], and Tango [33]. The results of their predictions were not unanimous (Table S1 in SI).

Since experimental data on the fragments were only published for *E. coli*, we verified our predictions based on their reported aggregation propensities. None of the predictors provided the classification results in good agreement with experimental data, which report R1, R3, and R5 of *E. coli* as capable of forming aggregates [9]. The best agreement was obtained for the R1 fragment (AmyloGram, PATH, and Waltz). The lack of agreement between computational and experimental results may partly come from the functional character of these sequences. All of the presented methods were trained mostly on fragments of pathological amyloids and their mutants, which somehow showed different characteristics from functional amyloids [34,35]. However, we believe that these methods are still sensitive enough to detect differences between highly similar fragments whose scarce point mutations are indicated as potentially leading to changing of the aggregation propensity. Therefore, we expected differences in amyloid aggregation of R4 fragments from *E. coli* and *S. enterica* bacteria, which we tested experimentally.

### 2.3. Experimental Analysis

Spectroscopic techniques (CD, ATR-FTIR, and FT-Raman) were used to study aggregation propensity of all *E. coli* and *S. enterica* fragments. These methods provide general information about the secondary structure and allow for monitoring of the fibrillization process [36,37,38,39]. Finally, we performed transmission electron microscopy (TEM) to analyze morphology of the selected fragments [40].

#### 2.3.1. Circular Dichroism

**Figure 2:**
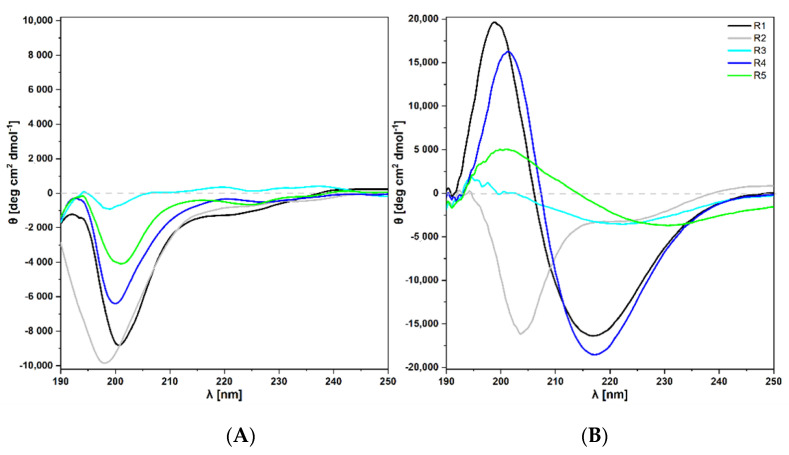
Far-UV CD spectra of CsgA fragments on the day of the dissolving. (**A**) Spectra for *E. coli* fragments, (**B**) Spectra for *S. enterica* fragments on the day of the dissolving (final peptide concentration 500 μM).

Circular dichroism (CD) spectroscopy was used to elucidate general characteristics of the secondary structure of the CsgA fragments. CD spectra of *E. coli* fragments are presented in Figure 2A. On the day of sample dissolving, for all *E. coli* fragments, a minimum of ca. 200 nm could be observed in all recorded spectra, which is characteristic of a random coil conformation. The spectra resemble the results presented for the whole CsgA, studied by Shu et al. 2012 [41]. The authors observed that CsgA initially exhibited random coil structure; however, after 13 days of incubation, it showed the presence of a β-sheet structure.

CD spectra of *S. enterica* fragments are depicted in Figure 2B. The spectrum of R1 fragment showed a single maximum at 198 nm and a single minimum at 217 nm, which were characteristic of the β-sheet conformation. Analysis of R2 fragment revealed a single minimum at 203 nm, which corresponded to the random coil. Fragment R3 displayed a maximum at 194 nm and a broad minimum at 220 nm, which indicated the presence of the β-sheet. R4 fragment showed a maximum at 201 nm and a single minimum at 216 nm, which was also characteristic of the β-sheet conformation. In case of R5 fragment, the broad maximum at 200 nm and broad single minimum at 230 nm could also be assigned to the β-sheet conformation.

In summary, CsgA fragments of *S. enterica* show two types of structures: β-sheet conformations can be assigned to fragments R1 and R3–R5, the random coil conformation is present in case of fragment R2. Shifting in peaks positions and a weak negative Cotton effect, observed especially for fragments R3 and R5, can be a consequence of the aggregation process occurring during the measurement. This was also observed with other techniques, as we discuss further.

The results of the secondary structure analysis, based on CD spectra, indicate that the rate of assuming the β-sheet conformation is higher in the fragments from *S. enterica* than in their counterparts from *E. coli*. While all *E. coli* fragments were still in the phase of the random coil structure, all potentially aggregating fragments of *S. enterica* showed β-sheet conformations on the day of dissolving. This process seemed most advanced for R1 and R4 fragments; however, the results for R5 and R3 fragments also indicated the onset of amyloid aggregation.

#### 2.3.2. ATR-FTIR

ATR-FTIR spectra in the wavenumber range of 1725–1590 cm^−1^ were used for a more advanced secondary structure analysis of the CsgA fragments.

**Figure 3:**
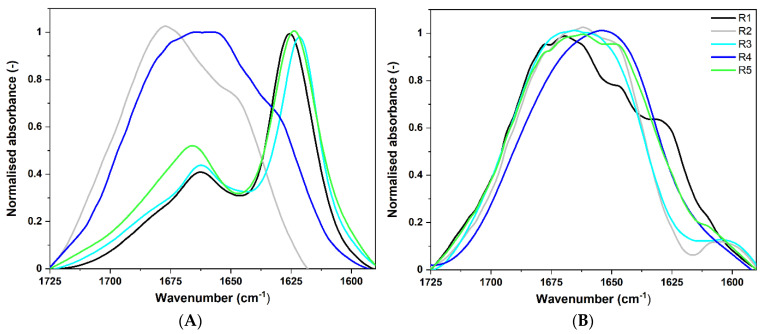
Normalized ATR-FTIR spectra of *E. coli* fragments in the wavenumber range of 1725–1590 cm^−^^1^ (Amide I), smoothed SG 35 (see Methods). (**A**) on the day of the dissolving (**B**) after one month of incubation at 37 °C. Peptide concentration was 500 μM.

We compared the spectra of *E. coli* fragments obtained on the day of dissolving (Figure 3A) with those after one month of incubation at 37 °C (Figure 3B). Fragments R1, R3, and R5, on the day of dissolving, showed the main band located below 1630 cm^−1^, which corresponds to cross-β amyloid architecture (Figure 3A). It indicated the presence of aggregates [37]. These repeating units were considered as highly amyloidogenic. Wang et al. showed that R1 and R5 fragments are critical for CsgA protein to form fibrils [9]. The analysis of the second derivative spectrum of R3 unveiled that R3 formed more rigid and ordered fibrils than R1 or R5. It was revealed by the location of its main negative peak at lower wavenumbers at c.a. 1621 cm^−1^, as well as the band width. For fibril forming fragments R1, R3, and R5, the Amide I band had an additional local maximum located at approximately 1665 cm^−1^, which is typically attributed to the parallel β-sheet structure that shows a high-frequency component between 1670–1660 cm^−1^ [42]. It can also be assigned to turn structures [43,44], as well as loops [45]. High absorbances in that loop–turn region are characteristic of parallel β-helix structure and observed, for example, in infrared spectra of HET-s [46] or PrP^Sc^ [47], which are known to adopt beta solenoid conformations.

In turn, the fragment R2 had a more complex spectral characteristic, with a broad local maximum at 1678 cm^−1^ assigned to β-turns. The shoulder at 1648 cm^−1^ was typical of a random coil [48]. ATR-FTIR spectrum of R2 lacked the β-sheet component below 1640 cm^−1^ and, due to its low absorbance below 1630 cm^−1^, we concluded that this fragment did not manifest aggregation properties. These observations are consistent with CD results (see Section 2.3.1) and with literature [49,50]. Fragments R2 and R4 from *E. coli’s* CsgA are considered incapable of self-assembly into ordered amyloid fibers in vitro; albeit, it is worth adding that Wang et al. [51] observed fibers with TEM experiments in which R2 or R4 were incubated at 2 mg/mL at room temperature for 5 days. As expected, peptide R4 under our experimental conditions did not manifest significant amyloidogenic properties. However, the secondary derivative in the range of 1725–1590 cm^−1^ (Figure S5 in SI) revealed the presence of β-sheet low frequency component at ca. 1628 cm^−1^, and high frequency component in the range of 1710–1690 cm^−1^, typical of anti-parallel β-sheet [42]. The absorbance of these sub-bands was relatively low.

After 30 days of incubation at 37 °C we observed that Amide I bands in ATR-FTIR spectra, registered for R1, R3, and R5 fragments of *E. coli*, broadened (Figure 3B). They lost spectral signatures typical of aggregates. Nevertheless, in the second derivative, the spectrum of R1 (Figure S6 in SI) was still clearly visible, leading to the conclusion that the R1 fragment remained partially aggregated. Moreover, all fragments, excluding R4, exhibited local minima at about 1693 and 1678 cm^−1^, assigned to antiparallel β-sheet and β-turns, respectively. This spectral characteristic is typical of oligomers [36,42,52]. In turn, Amide I bands in the ATR-FTIR spectra of R2 and R4 were dominated by the sub-band at around 1644 cm^−1^, assigned to random structures.

We concluded that the aggregates formed by R1, R3, and R5 fragments of CsgA from *E. coli* were not stable in time under studied conditions. This phenomenon can be caused by deamination of asparagine and glutamine residues present in the fragments. This non-enzymatic reaction leads to carboxylic acid derivatives, confirmed by higher absorption in the range of 1725–1710 cm^−1^. The process is known to occur during the incubation in vitro [53,54] and also during peptide synthesis [55]. Deamidation process is generally slow, but it can be strengthened by experimental conditions, such as increased temperature. In our case, two factors may have influenced the observed effect: incubation time (30 days) and temperature (37 °C). After three months of the incubation process, disintegration of *E. coli* fragments appeared. This observation is very interesting with regard to the fact that CsgA protein is a functional amyloid from an organism frequently co-existing with humans, and as such, its fibrils should not be as stable as those from pathological amyloids.

**Figure 4:**
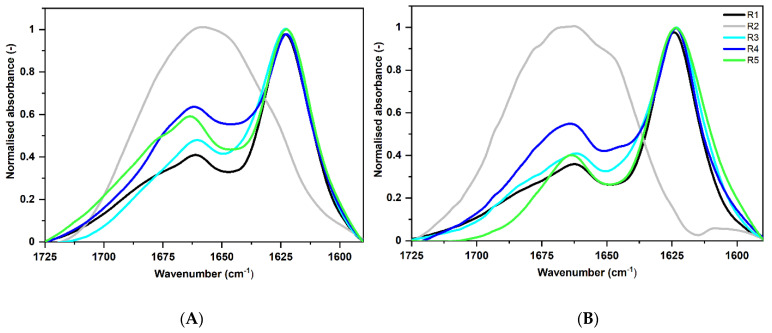
Normalized ATR-FTIR spectra of *S. enterica* fragments in the wavenumber range of 1725–1590 cm^−1^ (Amide I), smoothed with SG 35 (see Methods). (**A**) on the day of the dissolving (**B**) after month incubation at 37 °C. Peptide concentration was 500 μM.

Similarly, we compared the spectra of *S. enterica* fragments obtained on the day of dissolving (Figure 4A) with those measured after one month of incubation at 37 °C (Figure 4B). Spectra of fragments R1, R3, R4, and R5, directly after dissolving, showed a high intensive absorbance at about 1622 cm^−1^ (Figure 4A). This indicates the presence of long and rigid amyloid fibrils [36,56]. For the R2 fragment, a broad band located at about 1645 cm^−1^ in the Amide I, which is characteristic of disordered proteins [57], could be observed. After 30 days of incubation at 37 °C, we did not notice any significant changes in all studied ATR-FTIR spectra in the range of 1725–1590 cm^−1^ (Figure 4B). Contrary to *E. coli*, all *S. enterica* fragments maintained the same structures as they assumed directly after dissolving. This result indicates that CsgA fragments of *S. enterica* are more stable than those from *E. coli*. However, after 3 months of incubation at 37 °C, we observed that all fragments of *S. enterica* also disintegrated (data not shown).

The results from ATR-FTIR do not exactly match those obtained from CD, as we also observed here. ATR-FTIR experiments may speed up formation of amyloid fibrils due to interaction of peptides with the hydrophobic surface of the ATR accessory diamond. For example, early occurrence of amyloids in the fragments of *E. coli*, as shown by our ATR-FTIR experiments, could be related to the increased ratio of aggregation, not yet observed with CD at this stage.

A summary of the Amide I spectral analysis is presented in Table 1. The corresponding fragments are compared, including time effects. Locations of the most characteristic spectral components show propensity of each peptide to amyloid aggregation and other details on their secondary structures.

**Figure 5:**
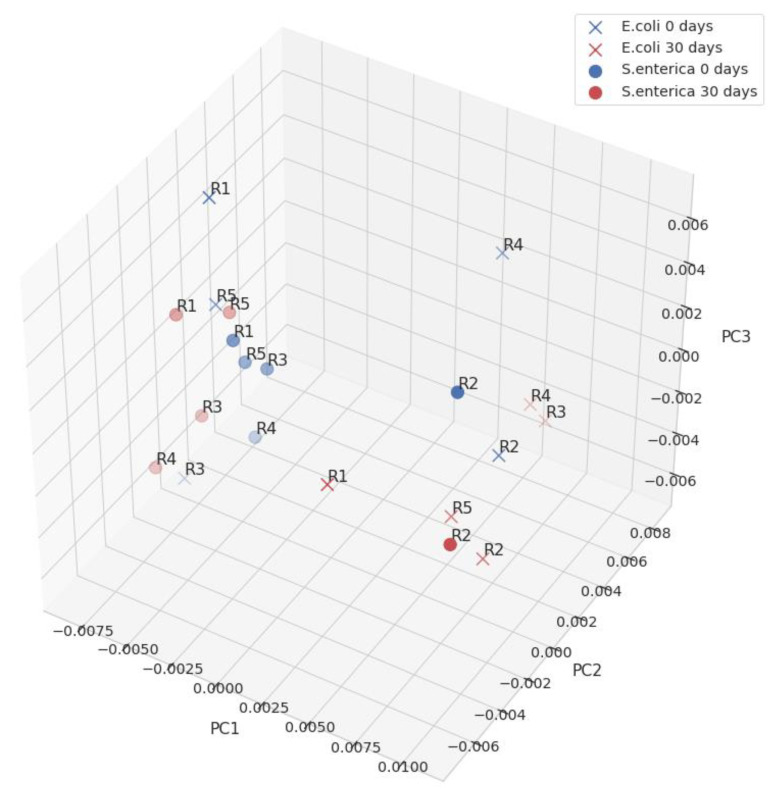
PCA plot for *E. coli* and *S. enterica*. Samples on the day of the dissolving and after 30 days of incubation at 37 °C. Points on the left side correspond to aggregates, on the right side to random structures.

We carried out principal component analysis (PCA) based on normalized ATR-FTIR spectra after application of SG 35, in the range of 1725–1590 cm^−1^ (see Methods). PCA analysis distinguished a class of aggregates in the set of studied peptides, based on the first three components (Figure 5 and Figure S1 in SI). Based on ATR-FTIR spectra of CsgA fragments, the loading plot of PC1 was obtained (Figure S2 in SI). It shows that the Amide I component at 1620 cm^−1^ strongly contributes to the separation of aggregates and non-aggregates. PC2 distinguished oligomers, due to high contributions of 1690, 1680, and 1630 cm^−1^. These features are characteristic of anti-parallel structures [52]. The results of PCA analysis matched those by a human expert, as presented in Table 1.

#### 2.3.3. FT-Raman

**Figure 6:**
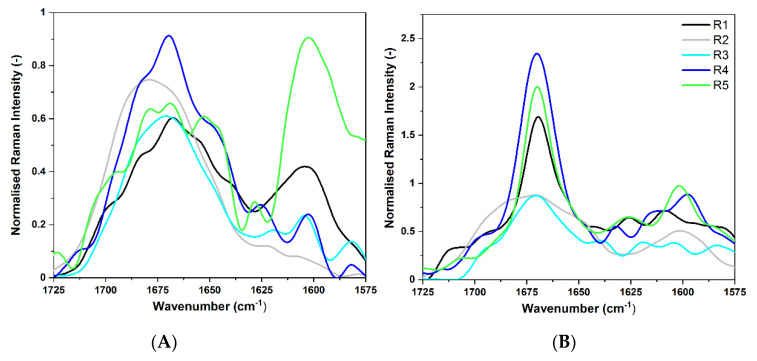
Normalized FT-Raman spectra of CsgA protein fragments, smoothed with SG 35 (see Methods), in the wavenumber range of 1725–1575 cm^−1^ (Amide I). (**A**) Spectra for *E. coli* fragments after 30 days of incubation at 37 °C, (**B**) Spectra for *S. enterica* fragments after 30 days of incubation at 37 °C. Peptide concentration was 500 μM.

For further structure analysis of the long-incubated CsgA fragments, FT-Raman spectroscopy was applied for R1–R5 fragments of *E. coli* and *S. enterica* after 30 days of incubation at 37 °C. This study can bring information complementary to ATR-FTIR regarding amyloid structures. FT-Raman technique is not frequently used for studying amyloids, although it can shed new light on structural analysis of aggregates. Results of FT-Raman spectra of CsgA fragments from *E. coli* and *S. enterica* are presented in Figure 6, including Amide I (1725–1575 cm^−1^), (second derivative spectra are available in Figure S7 in SI), and Amide III (1375–1185 cm^−1^) bands (Figure S3 in SI). The main drawback of studying peptides and proteins in aqueous solutions using FTIR spectroscopy is a strong water absorbance band at approximately 1635 cm^−1^ in Amide I band. Contrary to ATR-FTIR spectra, those from FT-Raman are usually analyzed in all these bands because there is no water interference in Amide I. Furthermore, different secondary structures of proteins have more observable differences in their amide III spectra [58]. Importantly, simultaneous analysis of two regions enables higher certainty of structure assignments. 

Raman results prove a partial deamidation of peptides corresponding to five imperfect repeating units of CsgA from *E. coli*. The FT-Raman spectra of *E. coli* subunits in the Amide I range are much more complex than the spectra of *S. enterica*, but in general, the Amide I band positions are characteristic of β structures [59]. Four fragments (R1, R3, R4, R5) of *E. coli* assumed a high percentage of β-sheet structures, which is indicated by the presence of a second derivative minimum at about 1670 cm^−1^ [60,61]. Bands in the range of 1695–1680 cm^−1^ are usually assigned to the β-turn structure [44]. However, disordered proteins also exhibit high contribution in that region [62]. Therefore, the R2 fragment of *E. coli* showed a broad Amide I band with the maximum at about 1691 cm^−1^, with an associated (shoulder) band at 1647 cm^−1^. The assignment of the sub-band at around 1645 cm^−1^ is debatable in literature, but in our opinion, it should be assigned to disordered structures. The appearance of additional Amide I mode at 1645 cm^−1^ is correlated with the strongly enhanced band of 1245–1255 cm^−1^ in the Amide III region (Figure S3 in SI), typically attributed to unordered structures.

Additionally, all spectra, except for R2, revealed higher intensities at about 1600 cm^−1^, due to surface-enhanced Raman spectroscopy (SERS) effect, which occurred upon the adsorption of the peptide on metal surfaces [63]. This broad band can be mainly attributed to ring modes of phenylalanine and tyrosine [64], but it also overlaps with other spectral features in that region, i.e., Amide II. The most intensive band at ~1600 cm^−1^ was observed for R1 and R5 fragments (Figure 6), which indicates the presence of the most rigid aggregates. The differences in its intensities can indicate various exposures of aromatic amino acids to the external environment, most probably caused by changes in tertiary structure of peptides during the aggregation process [65]. The analysis of Amide III (Figure 3A, Table S3 in SI) confirmed that all fragments possessed dominant β conformation but, in addition, revealed some differences in secondary structures between studied fragments. While fragments R1, R2, R4, and R5 had higher intensities near 1267 cm^−1^, which corresponded to the β-turns, the location of an amide III band at 1250 cm^−1^ obtained for R3 was typical of random and loose β structures. In all second derivative spectra in the range of 1375–1195 cm^−1^, the minimum at ~1230 cm^−1^ was present. It was most intensive in the spectrum of R5, and it could be assigned to the β-sheet structure [66].

In FT-Raman spectra of *S. enterica,* we observed that all peptides exhibited Amide I band maxima near 1670 cm^−1^, which is typical of β-structures [67,68]. As mentioned above, β-turns give an additional contribution to the spectra in the range of 1715–1675 cm^−1^. For the R2 fragment of *S. enterica,* the Amide I band was very broad with the full width at half maximum (FWMH) = 60 cm^−1^, indicating a complex structure (Table S4 in SI). The narrow (FWMH is 19 cm^−1^) and intensive Amide I band, as of R1, indicated the presence of well-ordered β-strands [38] (Figure 6B). The R3 fragment also exhibited a complex spectrum; however, the dominant maximum at 1671 cm^−1^ marked the signature of β-sheet conformation. All Amide bands of R3 consisted of more sub-bands than bands from other peptides (Figure 6B, Figure S3B in SI). Additionally, the spectrum had an increased intensity at about 1600 cm^−1^, which can be interpreted as a contribution from aggregates. The analysis of Amide III band confirms all above observations (see Figure S3, Table S3 in SI). The wavenumber range of 1375–1185 cm^−1^ was dominated by signatures typical of β structures. Fragments R4 and R5 exhibited intensive features at 1225 cm^−1^, which arose from β-sheet conformations [61,69].

A summary of the spectral analysis based on FT-Raman experiments is presented in Table 2. Locations of the most characteristic spectral components show propensity of each peptide to certain secondary structures.

Summarizing the results, FT-Raman spectroscopy showed that R1, R3, and R4 fragments of *E. coli* after 30 days of incubation contained a high number of β-sheet conformations, while R2 fragments had a dominant β-turn conformation. The presence of β-turns was also detected in R1, R4, and R5. Only R1 and R5 fragments showed some symptoms of amyloid aggregates. However, the Amide I band in FT-Raman spectrum registered for the R5 fragment of *E. coli* was typical of a random coil. It may indicate that fragment R5 formed less structured aggregates in comparison to the structure of R1. In the case of *S. enterica*, R1, R3–R5 fragments formed amyloid aggregates. However, R3 formed amyloid aggregates with additional contribution of other complex secondary structures. R2 had a complex non-aggregated secondary structure. Spectral signatures for this fragment are typical of a disordered conformation. R1, R4, and R5 represented a structure with dominant β-sheets.

Based on FT-Raman results, it is evident that all *S. enterica* CsgA sequence repeats show higher stability than corresponding fragments of *E. coli*. The fibril forming units exhibited intensive and narrow Amide I bands located at ~1670 cm^−1^, while in the case of *E. coli,* broad and complex FT-Raman signatures were observed in the range of 1725–1590 cm^−1^. FT-Raman findings are consistent with previously presented results from ATR-FTIR.

#### 2.3.4. Transmission Electron Microscopy

**Figure 7:**
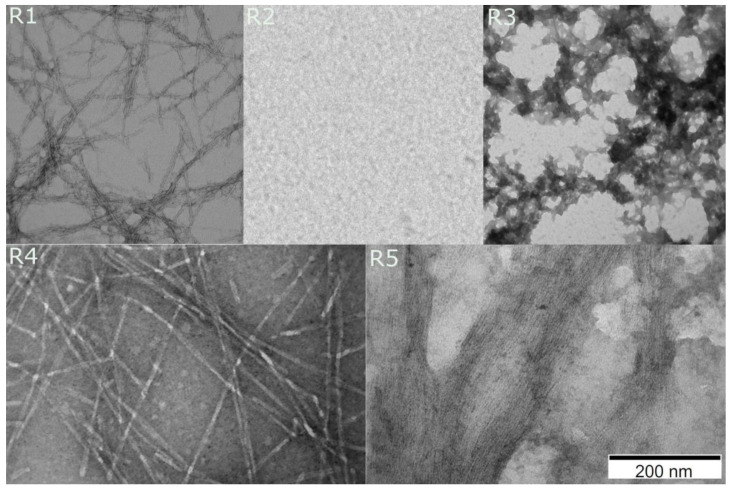
Electron micrographs of *S. enterica* fragments after one to seven days of incubation in 37 °C. Images registered at the magnification of 200 nm. Peptide concentration was 0.5 μM.

To observe the morphology and size of aggregates from *S. enterica* fragments, which have not been studied and published so far, we used transmission electron microscopy (TEM) [70,71]. Presence of fibrils was observed in cases of R1, R4, and R5 fragments. The observed fibrils were organized into rigid and high-ordered structures that ranged from 10 nm to 100 nm in diameter and from 500 nm to more than 1 μm in length. Interestingly, the morphology of fragment R3 differed from other fragments of *S. enterica*. The aggregates were composed of many connected oligomers/monomers. The observation matches the results from FT-Raman technique, which indicated a complex structure. This result shows that fragment R3 of *S. enterica* does not have as strong amyloid propensity as R1 and R5, similar to R3 of *E. coli* [49]. In turn, the micrographs of the R2 fragment did not reveal fibrils (Figure 7). These results are in agreement with ATR-FTIR and FT-Raman techniques, which also classified R2 as a non-aggregating fragment.

### 2.4. Comparative Analysis of R4 Fragments from S. enterica and E. coli 

Different prediction results from bioinformatics tools indicated that the R4 fragment may differ in the aggregation properties for sequences from *E. coli* and *S. enterica*. Our spectroscopy and microscopy results confirmed the computational prediction and demonstrated that R4 is the only fragment with different amyloid characteristics. Therefore, we carried out additional analyses regarding both sequences of R4. 

#### 2.4.1. ThT Assay

**Figure 8:**
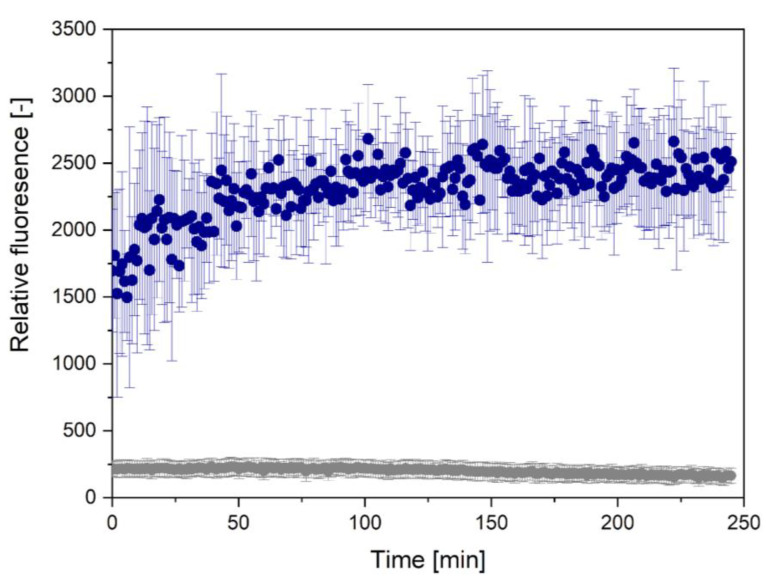
Time-dependent ThT fluorescence curves for R4 fragments. Here, grey dots represent *E. coli*, blue ones *S. enterica*. Peptide concentration was 500 μM.

To track the fibrillation kinetics, we performed a thioflavin-T (ThT) fluorescence assay [72]. The results are presented in Figure 8. A significant increase in the fluorescence emission was observed for *S. enterica*, which confirmed fibril assembly [73]. The fluorescence of R4 of *S. enterica* was about nine times higher than that of *E. coli* fragment. The lag phase was not observed there, which indicated rapid aggregation. The fibrillation steps of R4 of *S. enterica* indicated immediate elongation phase and saturation phase.

#### 2.4.2. Comparative Transmission Electron Microscopy Micrographs

**Figure 9:**
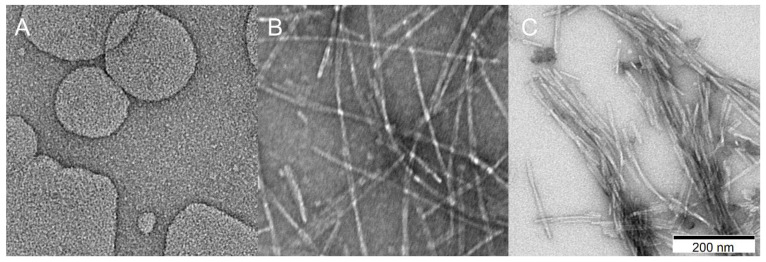
Comparison R4 fragments. (**A**) *E. coli* on the day of dissolving (**B**) *S. enterica* on day of dissolving (**C**) *E. coli* after 7 days of incubation in 37 °C. Images registered at the magnification of 200 nm. Peptide concentration was 0.5 μM.

The micrographs of R4 fragment from *E. coli,* measured on the day of the dissolving, did not reveal fibrils (Figure 9A). This result contrasted with the micrographs of *S. enterica*, which showed fibrils (Figure 9B). However, we made a very interesting observation regarding R4 fragment of *E. coli.* The fibrils of R4 were also observed for *E. coli*, but only after seven days of incubation at 37 °C (Figure 9C). Our results show that the R4 fragment has an amyloid propensity in both bacterial species; however, the aggregation process of isolated *S. enterica* fragments is much faster than that in *E. coli*.

## 3. Discussion

Bacteria produce biofilms to create an environment protecting them from adverse conditions using amyloid forming fibrils, such as CsgA curli protein. Understanding of the self-assembly propensity of CsgA can be based on evolutionary variability of imperfect repeats. The structural and functional nature of the CsgA protein is defined by R1–R5 fragments, representing non-perfect repeats of amino acid sequences. In this work we compared aggregation propensities of CsgA fragments in vitro from *E. coli* with those from *S. enterica*, not reported so far. The results may help in better understanding of the principles of curli aggregation and potential effects on human health, especially in the case of salmonellosis.

Based on previous publications on *E. coli* [9,49], it was expected that R1 and R5 fragments are the aggregation seeds, driving the amyloid propensity of CsgA. Fragment R3 is also capable of aggregation, while R2 and R4 are non-aggregating. Tendency to form amyloid fibrils is defined by certain gate-keeper residues, specific to each fragment. Generally, the repeat fragments are defined by the common motif: S-[X]5-Q-[X]-[G{Ec}/X{Se}]-[X]-[G{Ec}/X{Se}]-N-[X]-A-[X]3-Q, where {Ec} and {Se} indicate differences in motifs characteristic of *E. coli* and *S. enterica*, respectively. The discovered gate-keepers are located at the mutating positions, denoted here with [X].

One of those gate-keeper residues, observed in R1 fragment of *E. coli*, is a negatively charged glutamic acid (Q) at its position 7 and aspartic acid (N) at position 12. These positions are not mutated in *S. enterica*. It should also be noted that negatively charged aspartic acid (N) at position 23 is replaced by positively charged lysine (K) in *S. enterica*.

The non-amyloid nature of R2 is secured by the presence of glycine (G) at positions 13 and 17, as well as negatively charged aspartic acid (D) at position 15. This pattern is identical in both bacterial species, which suggests that R2 keeps its non-amyloid character also in *S. enterica*.

Fragment R3, which is a weak amyloid in *E. coli*, is slowed down in its amyloid susceptibility by two gate-keeper residues: aspartic acid (D) at positions 4 and 17. One of these residues is mutated in *S. enterica*—aspartic acid at position 4 is replaced by glutamic acid (D4E). This substitution should lead to stronger amyloid propensity in R3 from *S. enterica* when compared to its counterpart from *E. coli*.

An even greater change concerning gate-keeper residues appears in the R4 fragment. Here, aggregation in *E. coli* is slowed down by glycine (G) at position 13 and aspartic acid (D) at position 17. Both positions are substituted in *S. enterica*, glycine by alanine (G13A) and aspartic acid by glutamic acid (D17N). This substitution may lead to increased amyloid propensity in R4 from *S. enterica*.

Strong amyloid propensity of R5 from *E. coli* was assigned to negatively charged glutamic acid (N) residues at positions 4 and 12 and histidine (H) at position 17. Fragment R5 in *S. enterica* has two substitutions at these positions; negatively charged aspartic acid is replaced by uncharged methionine (N4M) and positively charged histidine by negatively charged aspartic acid (H17N). The substitutions of gate-keeper residues could affect the amyloid nature of R5.

We studied self-aggregation and secondary structures of the repeat fragments of *S. enterica* and *E. coli* and comparatively analyzed their potential effects on these proteins in a bacterial biofilm. Different methods were applied, including bioinformatics prediction, ATR-FTIR and FT-Raman spectroscopy techniques, circular dichroism, and transmission electron spectroscopy.

Bioinformatics predictors were not unanimous in their results and, unfortunately, not very accurate when confronted with reported results from experimental studies. This could have been due to the functional role of CsgA. Functional amyloids are scarcely represented in reference datasets on which computational predictors of amyloids are based. Therefore, a statistical sequence profile of functional amyloids is most probably significantly different from that of a pathological amyloid. This conjecture comes from different structural details of the two classes of amyloids, their different temporal characteristics, and different stability and controllability by environmental conditions and interactions. However, currently available methods are not totally useless regarding functional amyloids; they are capable of guiding a more informed search. In our studies, bioinformatic method AmyloGram predicted a different amyloid propensity of R4 fragments from *E. coli* and *S. enterica*, indicating that non-amyloid R4 from *E. coli* changes its nature in *S. enterica*, where it may be aggregating. The analysis of mutations in its gate-keeper residues supported this possibility. Although the R4 fragment of *E. coli* was previously reported as non-aggregating in similar conditions [9], there were no studies of R4 from *S. enterica*.

The clue from computational prediction was confirmed in our experiments—R4 from *S. enterica* turned out to be strongly aggregating. ThT kinetic studies showed very fast aggregation of R4 from *S. enterica*, in which we were not able to observe a lag phase. It contrasted with the results of the ThT study of R4 from *E. coli*, which did not show aggregation. The results were consistent with ATR-FTIR studies, showing more aggregates in R4 from *S. enterica* and the prevalence of random coils in *E. coli*. However, our TEM studies, taken after 7 days of incubation, showed that the initial lack of aggregates of R4 from *E. coli* did not reflect its true nature. R4 also forms amyloid fibrils, but the process is much slower than in *S. enterica*. Aggregation of R4 was also reported in [51], where its concentration was much higher than typically used in such studies, which indicated some amyloid propensity of the peptide. Nevertheless, after 30 days of incubation, the aggregates of R4 from *E. coli* disintegrated. The general structure was random coil, however, the presence of oligomers could not be excluded. Stability of R4 from *S. enterica* turned out differently—the aggregates were unchanged after 30 days, as indicated by ATR-FTIR and FT-Raman studies.

We note that, in aggregation prone peptides, charged residues were observed mostly in terminal parts. Additional arguments for the importance of the charge distribution can be found in the change of aggregation propensity of the R4 fragment, observed from our study. In its sequence derived from *E. coli*, two charged amino acids are located outside the terminal or linker region (6K and 17D). However, in its strongly aggregating counterpart form *S. enterica,* they are no longer present. The replacement of amino acids at positions 17 and 21 leads to the change in charge distribution. This, alongside the loss of charge at position 6, leads to the structure with all charged amino acids at the same side of the folded peptide, and outside the core of the predicted β-arch. Combined with the loss of gate-keeper residues, it could be the key reason for the different amyloid propensity.

The experimental techniques confirmed self-aggregation of R1, R3, and R5 fragments for both species, as previously reported for *E. coli*, and now also shown by ATR-spectra and TEM micrographs for *S. enterica*. We also observed a much weaker aggregation propensity of R3 than R1 and R5 fragments, as previously reported by other studies for *E. coli* [49]. The ratio of aggregation was significantly lower for fragments of *E. coli*, as obtained from CD spectra and ThT-measurements. However, disintegration of amyloid fibrils in *E. coli* proceeded faster, as shown by ATR-FTIR and FT-Raman techniques for the fragments after 30 days of incubation.

The multitude of techniques applied in our studies also revealed other more subtle details regarding the aggregation processes and secondary structures of the repeating fragments, indicating also the presence of more complex structures formed of some fragments, as well as their evolution over time. All spectroscopy techniques confirmed the presence of β-harpin structure of monomers in general confirmation of CsgA fragments. All fragments, except R2, exhibited signatures of turns and β-sheet structures in vibrational techniques. These results are in agreement with previously published computational models of CsgA [74,75] and ssNMR structure [76].

We also showed that FT-Raman can be used as a complementary technique to infrared spectroscopy in amyloid studies [77]. It provides information about secondary structure and, additionally, about tertiary structure—revealing exposure of aromatic amino acids to the external environment. The undoubted advantage of Raman spectroscopy is the fact that the analysis of the well-resolved Amide III band provides complementary structural information to the Amide I. Using the FT-Raman technique, we could observe that R3 fragment of *S. enterica* had more complex structures than other fragments of CsgA. More sub-bands were present in the second derivative of R3 spectra than in those of other fragments. TEM images confirmed that the fibrils of R3 were morphologically distinct. The morphological differences can be caused by location of charged amino acids in the sequence. The R3 fragment contains additional positively charged amino acids in the linker region and negatively charged aspartic acid residue at position 17.

Our studies showed that amyloid structures of CsgA repeats are more rapidly formed and more stable in *S. enterica* than those in *E. coli*, which has not been demonstrated so far. This result seems to be in accordance with in vitro aggregation of different curli variants, where the self-assembly of CsgA from *E. coli* is slower than that from *S. typhimurium* [11]. This phenomenon might be related to the general lifestyle of the Salmonella genus, where biofilm formation seems to be an important long-term colonization strategy [78]. Quicker self-assembly of CsgA could provide an advantage towards the prolonged infection.

Although there are no relevant reports concerning human amyloid diseases associated with salmonellosis, some clues could be derived from animal studies. As reported in [79], bacterial infection with *Salmonella Typhimurium* of the brains of transgenic 5XFAD mice resulted in rapid seeding and accelerated β-amyloid deposition, which closely colocalized with the invading bacteria. This finding could support a hypothesis that β-amyloid may play an immuno-protective role against bacterial infections and drive amyloidosis as a side effect. However, another mechanism may also be in play—a cross-talk between amyloid curli in bacterial biofilm and β-amyloid peptides where interactions of human proteins with bacterial curli accelerate formation of pathological aggregates. Therefore, understanding of amyloid propensity of Salmonella curli could be instrumental in studying aspects of human amyloid diseases.

Another important novelty in our studies is simultaneous use of a combination of several different experimental techniques. This approach enabled comparing different aspects revealed by each of the methods. In particular, FT-Raman spectroscopy was applied, which is very infrequently used in amyloid research. We also studied temporal changes in amyloid characteristics, regarding the curli from both species, not reported so far.

Further studies are required to shed more light on the surprising efficiency of self-assembly of CsgA produced by *S. enterica*, especially, effects of the sequence variability on the whole protein characteristics in vivo. The results would contribute significantly to better understanding of the curli aggregation.

## 4. Materials and Methods

### 4.1. Sample Preparation

CsgA *S. enterica* and *E. coli* fragments sequences were provided by CASLO (CASLO ApS, Denmark) (Table S4 in SI). Additionally, fragments: R2, R5 and partially R3 of *E. coli* of strain K12, were synthesized “in-house”. The synthesis was carried out with an automated solid-phase peptide synthesizer (Liberty Blue, CEM) using rink amide AM resin (loading: 0.59 mmol/g) (Table S5, Figure S6 in SI). Fmoc deprotection was achieved using 20% piperidine in DMF for 1 min at 90 °C. A double-coupling procedure was performed with 0.5 M solution of DIC and 0.25 M solution of OXYMA (1:1) in DMF for 4 min at 90 °C. Cleavage of the peptides from the resin was accomplished with the mixture of TFA/TIS/H2O (95:2.5:2.5) after 3 h of shaking. The crude peptide was precipitated with ice-cold Et2O and centrifuged (9000 rpm, 15 min, 4 °C). Peptides were purified using preparative HPLC (Knauer Prep) with a C18 column (Thermo Scientific, Hypersil Gold 12 μ, 250 mm × 20 mm) with water/ACN (0.05 TFA) eluent system. The purity of synthesized peptides was in the range between 95% and 99.6%. A sample of each peptide was dissolved in 490 μL of 0.01 M NaOH and vortexed for one minute. Then, 450 μL of phosphate-buffered saline (PBS) pH 7.2 was added, followed by 60 μL of Milli-Q^®^ (Merck & Co. Inc., USA) water, pH 6.9. The final concentration of the aliquot was about 500 μM, pH 7.4. To obtain monomers, each sample was filtered through a 0.2 μm PVDF syringe filter (Table S2 in SI, prepared based on the MIRRAGGE protocol [80]).

Initial monomerization of aggregates is a necessary step; however, it may affect the results with regard to their full validity when they are extrapolated to actual behavior of the protein. The lack of initial protofibrils, which constitute transient pre-fibrillar intermediates, may affect the pathway of further aggregation process. The filtration method lowers the effective concentration of the peptides.

### 4.2. Bioinformatic Analysis

The aggregation propensity of studied peptides was assessed using nine bioinformatics methods: AmyloGram [24], PATH [25], Pasta2.0 [28], Waltz [29], AmylPred2 [30], FoldAmyloid [31], MetAmyl [32], Tango [33], and ArchCandy [19]. Each predictor was used with its default parameters. Pairwise alignments of corresponding regions in *E. coli* and *S. enterica* were visualized using Jalview software [81].

### 4.3. Circular Dichroism (CD)

CD spectra were recorded on JASCO J-815 at 20 °C between 250 and 190 nm in PBS buffer pH = 7.2 with the following parameters: 0.2 nm resolution, 1.0 nm bandwidth, 20 mdeg sensitivity, 0.25 s response, 50 nm/min scanning speed, and 0.02 cm cuvette path length. The sample concentration was 500 μM. The CD spectra of the solvent alone was recorded and subtracted from the raw data. The CD intensity is given as mean residue molar ellipticity (θ [deg × cm^2^ × dmol^−1^]). Spectra were smoothened and plotted using Origin 2020b software.

### 4.4. Attenuated Total Reflectance—Fourier-Transform Infrared (ATR-FTIR)

FTIR studies were performed using Nicolet 6700 FTIR spectrometer (Thermo Scientific, USA) with ATR accessory and heated diamond top-plate (PIKE Technologies), continuously purged with dry air. Each sample of 10 μL of peptide aqueous solution was dropped directly on the diamond surface and allowed to dry out. All ATR-FTIR spectra were obtained in the range of 3600–400 cm^−1^. For each spectrum, 512 interferograms was co-added with 4 cm^−1^ resolution at constant temperature 22 °C [71.6 F]. Directly before sampling, the background spectrum of diamond/air was recorded as a reference (512 scans, 4 cm^−1^). We used 500 μM concentration, which was essential to obtain a good signal-to-noise ratio. The raw data are shown in Tables S7 and S8 in SI.

### 4.5. FT-Raman

Raman spectra were carried out using a Nicolet NXR 9650 FT-Raman spectrometer with MicroStage extension equipped with Nd:YVO4 laser (1064 nm, 500 mW) as an excitation source and InGaAs detector. A drop of 10 μL of each sample was deposited on the gold surface and dried under laser irradiation. All FT-Raman spectra were acquired in the range of 3700–0 cm^−1^ with 4 cm^−1^ resolution by averaging 1024 scans. The raw data are shown in Table S9 in SI.

### 4.6. Spectral Analysis

The spectra were analyzed using OriginPro 2020b (OriginLab Corporation, USA). The analysis included spectra baseline correction, smoothing using the Savitzky–Golay filter (polynomial order 2, widow size 35, SG 35) [82], normalization of spectra relative to Amide I band (ATR-FTIR), or deformation vibrations of CH_2_ group, at 1450 cm^−1^ (FT-Raman).

PCA was performed on the second derivative of the Amide I region of the spectra (1725–1590 cm^−1^) using Scikit-learn Python package [83]. Matplotlib [84] Python package was used for visualization.

### 4.7. Thioflavin T (ThT) Fluorescence Assay

The fluorescence of each well was read by a microplate reader CLARIOstar, as well as BMG LABTECH at 25 °C with 30 s shaking every 58.8 s during 244.85 min measurements. The samples containing 10 μL of 500 μM peptide and 90 μL of 500 μM ThT solution were mixed in a 96-well plate. The excitation wavelength was set at 440 nm and emission at 480 nm. Each group of experiments contained six parallel samples, and the data were averaged after measurements.

### 4.8. Transmission Electron Microscopy (TEM)

Imaging was performed using a transmission electron microscope Hitachi H-800 (Hitachi HighTech, Japan) on accelerating voltage of 150 kV. Negative stained samples were prepared by applying a 4 μL drop of solution containing 0.5 μM peptide in water on glow discharged carbon on copper grid (Agar S160, Agar Scientific Ltd, United Kingdom). After 1 min of adhesion, an excess of the material was blotted, and 2% uranyl acetate was applied for 1 min before blotting. The samples were allowed to dry under normal conditions for at least 1 h.

## Figures and Tables

**Table 1:** Secondary structure assignments of the studied peptides with sequences from *E. coli* and *S. enterica* on the basis of Amide I (ν(C=O) 80%, ν(NH) 20%) band in ATR-FTIR spectra. The results are from experiments on the day of dissolving and after incubation for 30 days at 37 °C. Band positions (cm^−1^) are presented, along with tentative assignments based on the most intense local minima of the second derivatives.

	After Dissolving		30 Days	
	cm^−1^	Assignment	cm^−1^	Assignment
***E. coli***				
R1	1626	aggregates	1679	turns
R2	1667	turns	1646	random
R3	1621	aggregates	1679	turns
R4	1654	random	1642	random
R5	1624	aggregates	1646	random
***S. enterica***				
R1	1626	aggregates	1679	turns
R2	1645	random	1646	random
R3	1622	aggregates	1623	aggregates
R4	1622	aggregates	1623	aggregates
R5	1622	aggregates	1624	aggregates

**Table 2:** Main band positions of Amide I in FT-Raman spectra of studied peptides in aqueous solution after 30 days of incubation at 37 °C. Band positions (cm^−1^) along with tentative assignments based on the most intensive local minima of the second derivatives.

	30 Days	
	cm^−1^	Assignment
***E. coli***		
R1	1668	β-sheet
R2	1691	turns
R3	1667	β-sheet
R4	1669	β-sheet
R5	1668, 1680	β-sheet/turns
***S. enterica***		
R1	1670	β-sheet
R2	1670, 1697	β-sheet/turns
R3	1671	β-sheet
R4	1670	β-sheet
R5	1670	β-sheet

## Supplementary Materials

The following are available online at https://www.mdpi.com/article/10.3390/ijms22105127/s1, **SI:** Table S1: Amyloidogenicity prediction results. Where 0 denotes non-amyloid, 1 stands for amyloid., Table S2: MIRRAGGE, Figure S1: Scree plot of PCA from ATR-FTIR spectra., Figure S2: Loading plot of PCA analysis as is resulted from the ATR-FTIR data., Figure S3: Normalized FT-Raman spectra of CsgA protein fragments with the second derivatives spectra smoothed 2 times with SG 35 in the wavenumber range of 1375–1185 cm^−1^ (Amide III). (A) Spectra for *E. coli* fragments after 30 days of incubation at 37 °C, (B) Spectra for *S. enterica* fragments after 30 days of incubation at 37 °C., Table S3: Main band positions of Amide III in FT-Raman spectra of studied peptides in aqueous solution after 30 days of incubation at 37 °C. Band positions (cm^−1^) along with tentative assignments based on the minima of the second derivatives. Bold values indicate the most intensive local minima., Table S4: Full width at half maximum (FWHM) of Amide I in the FT-Raman., Table S5: Peptides analytical data purchased from CASLO., Table S6: Peptides analytical data synthesized “in house”., Figure S4: Analytical HPLC chromatograms of “in house” studied peptide., Table S7: Raw ATR-FTIR spectra of *E. coli* fragments in the range of 3600–900 cm^−1^, Table S8: Raw ATR-FTIR spectra of *S. enterica* fragments in the range of 3600–900 cm^−1^, Table S9: Raw FT-Raman spectra of *E. coli* fragments in the range of 3600–600 cm^−1^, Figure S5: ATR-FTIR second derivatives spectra of *E. coli* fragments in the wavenumber range of 1725–1590 cm^−1^, smoothed twice with SG 35 (see Methods). (A) on the day of the dissolving (B) after month incubation at 37 °C. Peptide concentration was 500 μM, Figure S6: ATR-FTIR second derivatives spectra of *S. enterica* fragments in the wavenumber range of 1725–1590 cm^−1^, smoothed twice with SG 35 (see Methods). (A) on the day of the dissolving (B) after month incubation at 37 °C. Peptide concentration was 500 μM, Figure S7: FT-Raman second derivatives spectra, smoothed twice with SG 35 (see Methods), in the wavenumber range of 1725–1575 cm^−1^. (A) Spectra for *E. coli* fragments after 30 days of incubation at 37 °C, (B) Spectra for *S. enterica* fragments after 30 days of incubation at 37 °C. Peptide concentration was 500 μM.
