# Supplementary material for: Variability of Amyloid Propensity in Imperfect Repeats of CsgA Protein of Salmonella enterica and Escherichia coli
Source: Int J Mol Sci. 2021 May 12;22(10):5127. doi: 10.3390/ijms22105127 (PMC8151669; doi:10.3390/ijms22105127)
Supplement: Supplementary file 1 [file ijms-22-05127-s001.zip › ijms-1197308-supplementary.pdf]

Supplementary materials

Variability of amyloid propensity in imperfect repeats of CsgA protein of *Salmonella enterica* and *Escherichia coli*

Natalia Szulc [1,2], Marlena Gąsior-Głogowska [1], Jakub W. Wojciechowski[1], Monika Szefczyk [3], Andrzej M. Żak [4], Michał Burdukiewicz [5,6,7]*, Malgorzata Kotulska [1]*

[1] Department of Biomedical Engineering, Faculty of Fundamental Problems of Technology, Wroclaw University of Science and Technology, Wybrzeże Wyspiańskiego 27, 50-370 Wroclaw, Poland;

[2] LPCT, CNRS, Université de Lorraine, F-54000 Nancy, France

[3] Department of Bioorganic Chemistry, Faculty of Chemistry, Wroclaw University of Science and Technology, Wybrzeże Wyspiańskiego 27, 50-370 Wrocław, Poland;

[4] Electron Microscopy Laboratory, Faculty of Mechanical Engineering, Wroclaw University of Science and Technology, Wybrzeże Wyspiańskiego 27, 50-370 Wroclaw, Poland

[5] Clinical Research Centre, Medical University of Białystok, Jana Kilińskiego 1, 15-089 Białystok, Poland;

[6] Institute of Biochemistry and Biophysics, Polish Academy Sciences, 02-106 Warsaw, Poland;

[7] Faculty of Natural Sciences, Brandenburg University of Technology Cottbus-Senftenberg, 01968 Senftenberg, Germany;

Table S1. Amyloidogenicity prediction results. Where 0 denotes non-amyloid, 1 stands for amyloid.

Organism	Fragment	AmyloGram	PATH	PASTA2.0	Waltz	AmylPred2	FoldAmyloid	MetAmyl	ArchCandy*
E. Coli	**R1**	1	1	0	1	0	0	0	1
	**R2**	0	0	0	0	0	0	1	1
	**R3**	0	0	0	0	0	0	0	1
	**R4**	0	0	0	0	0	0	1	1
	**R5**	0	0	0	0	0	0	1	1
S. Enterica	**R1**	1	1	0	1	1	1	1	1
	**R2**	0	0	0	0	0	0	1	1
	**R3**	0	0	0	0	0	0	0	0
	**R4**	1	0	0	0	0	0	1	1
	**R5**	0	0	0	0	0	0	1	1

* β-arch motif predictor

Table S2. MIRRAGGE

**Sample details**						
Organism/Peptide Sequence		UniProt code (residues)	pI	GRAVY	Extinction coefficient [A 280, 0.1%(w/v)]*	MW from chemical composition (Da)
STLSIYQYGSANAALALQSDARK		P0A1E7	10.02	-0.19	2560	2427.67
SETTITQSGYGNGADVGQGADN		P0A1E7	3.54	-0.87	1280	2141.12
STIELTQNGFRNNATIDQWNAKN		P0A1E7	9.85	-1.08	5980	2634.82
SDITVGQYGGNNAALVNQTASD		P0A1E7	3.71	-0.35	1280	2194.27
SSVMVRQVGFGNNATANQY		P0A1E7	11.12	-0.29	1280	2042.24
SELNIYQYGGGNSALALQTDARN		P28307	6.63	-0.06	2560	2454.61
SDLTITQHGGGNGADVGQGSDD		P28307	3.71	-0.85	n.a	2100.08
SSIDLTQRGFGNSATLDQWNGKN		P28307	9.85	-0.96	5690	2508.66
SEMTVKQFGGGNGAAVDQTASN		P28307	6.66	-0.5	n.a	2168.3
SSVNVTQVGFGNNATAHQY		P28307	9.57	0.36	1280	1993.1
Source (supplier, catalogue No. or reference)					CASLO and "in house"	
N-terminal modification					---	
C-terminal modification					---	
Internal modifications					---	
Other modifcations					---	
Purity (%)					≥95%	
**Purification** (If applicable)	Chromatography techniques				RP-HPLC	
	oncentration of stock solution (M, mg/ml				4 mg/ml	
	Storage/Reconstitution buffer				water/ACN	
	Method of protein quantification				UV/VIS	
	Storage conditions				Lyophilized	
	Additional key information				---	
**Sample quality control**						
**Polishing step**	Immediately before the aggregation assay				---	
	Concentration (M, mg/mL)				---	
	Method quantification				---	
Method of detection		CD	ATR-FTIR	FT-Raman	ThT	TEM
**Aggregation assay**	Equipment details	JASCO J-815	Nicolet 6700 spectrometer	Nicolet NXR 96	CLARIOstar Plus	Hitachi H-800
	Measurement parameters	resolution,	512sc, 4cm	1024 scans, 4cm-1	emission 480 nm	accelerating voltage of 150 kV
	Plate/cuvette reference	OH, 50 mM P	---	---	water + ThT	---
	Assay volume	30 µL	10 µL	10 µL	10 µL	4 µL
	Evaporation control method	---	---	---	---	---
	Seeding details (if applicable)	---	---	---	---	---
	Shaking – Intensity	---	---	---	---	---
	Shaking – Shaking mode	---	---	---	yes	---
	Shaking – Frequency	---	---	---	every 58.8 second	---
	Beads – Reference	---	---	---	water + ThT	---
	Beads – Number/assay	---	---	---	3 repats	---
	Temperature of measurement (ºC)	20				
	Temperature of incubation (ºC)	37				
	Concentration (M, mg/mL)	500 µM	500 µM	500 µM	500 µM	0.5 µM
	Aggregation buffer and aditives	0.1 M NaOH, 50 mM PBS, pH 7.4				
	Measurement frequency	0 days	0, 30 days	30 days	0 days	7 days
	Assay duration	5 minutes	1.5 hour	1 hour	4 hour 10 minutes	1 hour
	Plate/cuvette setup	---	---	---	---	---
	Additional key steps	---	---	---	---	---

*Calculated based on: http://bestsel.elte.hu/extcoeff.php [Extinction coefficient at 205 nm, concentration units: $M^{-1}cm^{-1}$

Blue color corresponds to *S. enterica*, black to *E.coli*.

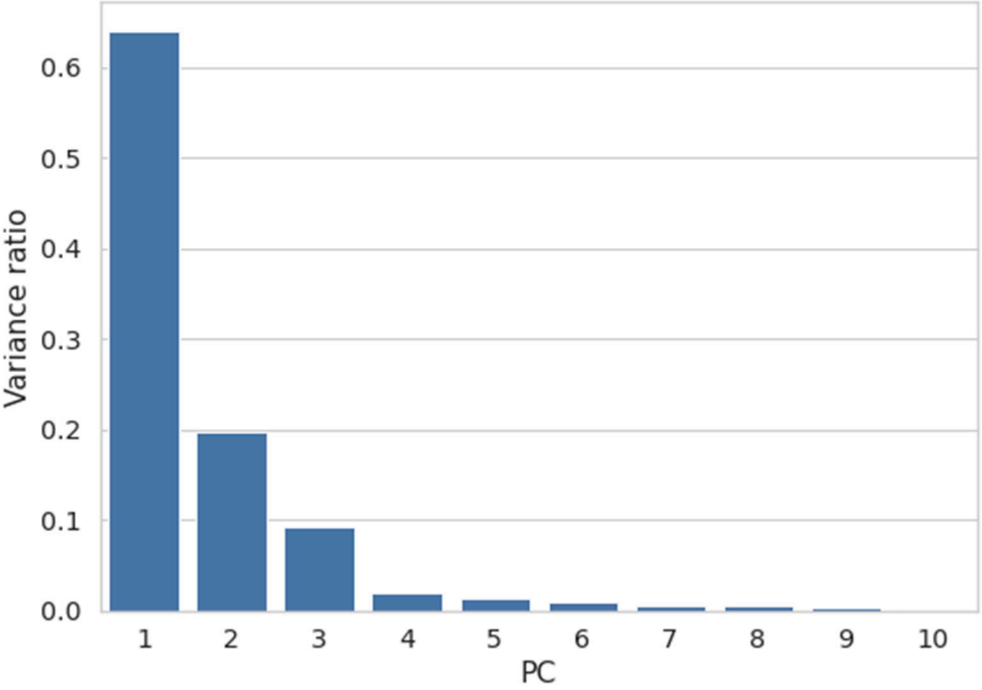

Figure S1. Scree plot of PCA from ATR-FTIR spectra.

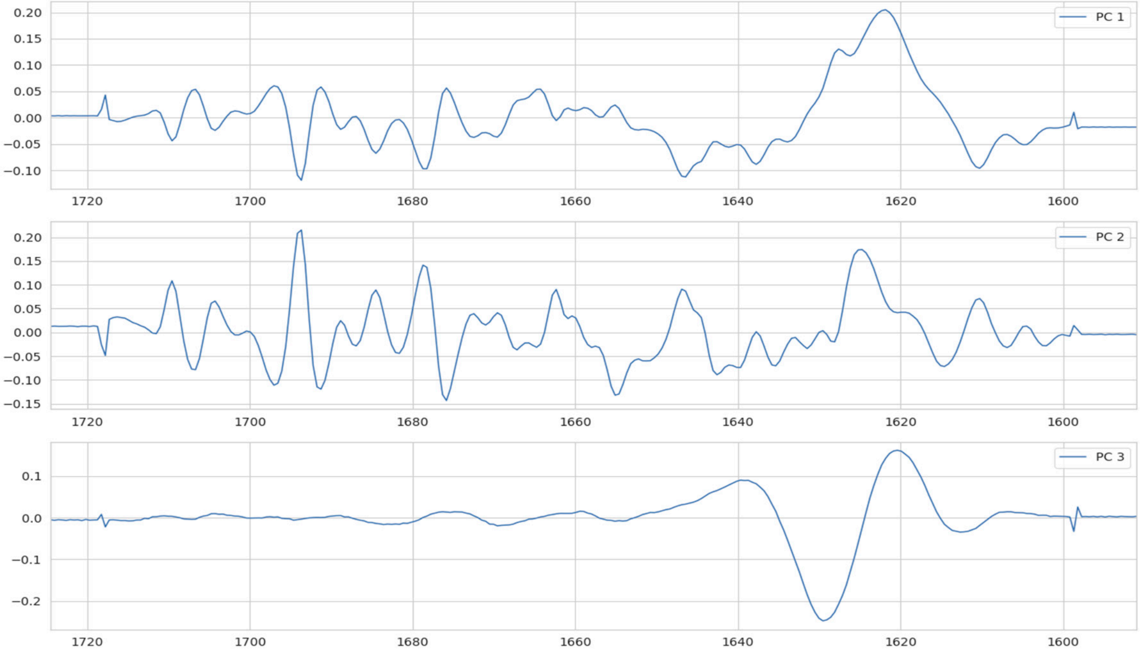

Figure 2. Loading plot of PCA analysis as is resulted from the ATR-FTIR data.

Loading scores describe which frequencies contribute to a given principal component (Fig. 2). It is important to highlight that the higher the number of a principal component the less variability of data it explains and the PC becomes less informative in data classification. Therefore, PC1 captures more information and affects the cluster separation more than PC2 or PC3. The ratios of explained variance are presented on a scree plot (Fig. 1).

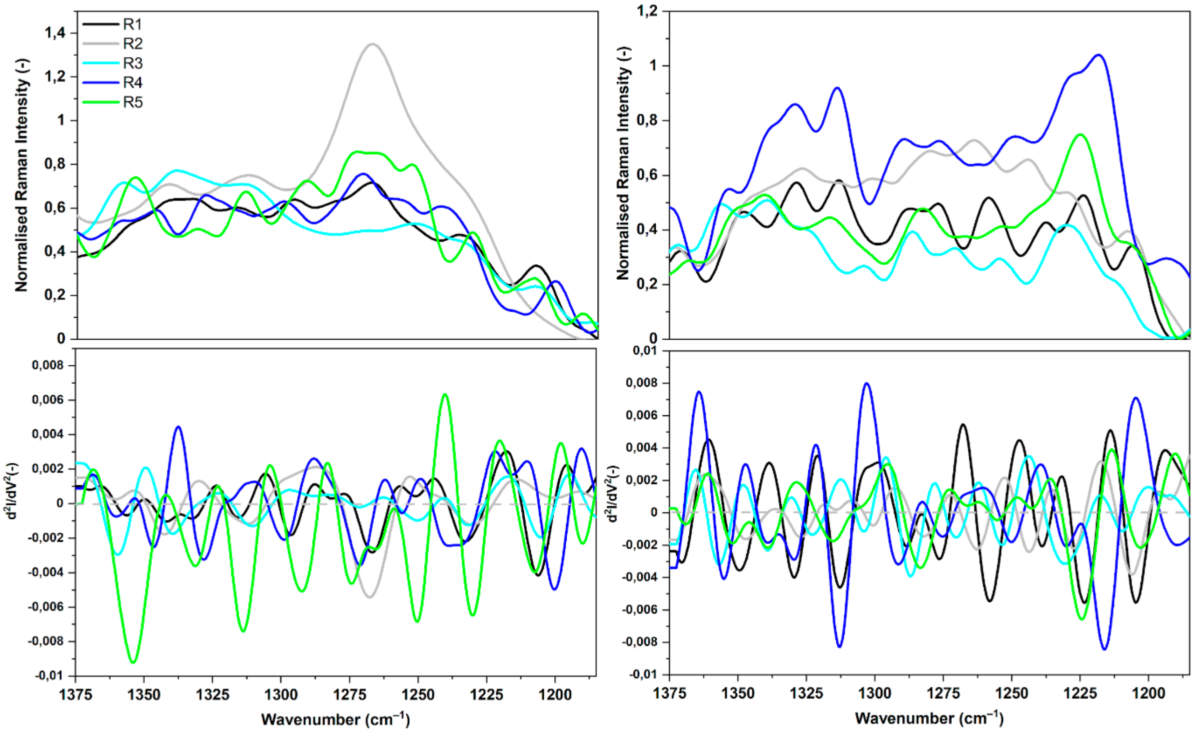

(A) **(B)**

Figure 3. Normalized FT-Raman spectra of CsgA protein fragments, with the second derivative spectra, smoothed 2 times with SG 35, in the wavenumber range of 1375-1185 cm^{-1} (Amide III). (**A)** Spectra for *E. coli* fragments after 30 days of incubation at 37 °C , (**B)** Spectra for *S. enterica* fragments after 30 days of incubation at 37 °C.

Table S3. Main band positions of Amide III in FT-Raman spectra of studied peptides in aqueous solution after 30 days of incubation at 37 °C. Band positions (cm^{-1}) along with tentative assignments based on the minima of the second derivatives. Bold values indicate the most intensive local minima.

Structure	H_2/alfa	α-helix	turns	random	β-sheet	β-sheet/AA	AA
E. coli							
R1	1315	1296	1266			1233	**1206**
R2	1311		**1267**			1230	
R3	**1310**			1250		1231	1205
R4		1299	1270		1236		**1201**
R5	1313	1292	1274	1251		1229	1207
S. enterica							
R1	**1313**	1288	1276	**1258**	1239	1223	1204
R2	1300	1282		1262	1244	1227	**1205**
R3	1304	**1286**	1270	1253		1230	1209
R4	**1313**	1292	1275			1230	**1215**
R5	1315	1283		1255		**1224**	1202

Where AA corresponds to aromatic amino acids.

Table S4. Full width at half maximum (FWHM) of Amide I in the FT-Raman spectra.

Structure	WMH [cm^{-1}]
E. coli	
**R1**	**58**
**R2**	**53**
**R3**	**49**
**R4**	**52**
**R5**	**64**
S. enterica	
**R1**	**19**
**R2**	**60**
**R3**	**35**
**R4**	**23**
**R5**	**18**

Table S5. Peptides analytical data purchased from CASLO.

Name	Sequence	Formula	$M_{t.}$[1]	M_{MS}[2]	HPLC $t_{ret.}$[3] [min]
R1	STLSIYQYGSANAALALQSDARK	$C_{105}H_{171}N_{31}O_{35}$	2428.71	2429.16	8.840[8]
R2	SETTITQSGYGNGADVGQGADN	$C_{85}H_{133}N_{27}O_{38}$	2142.15	2142.93	5.583[9]
R3	STIELTQNGFRNNATIDQWNAKN	$C_{112}H_{176}N_{36}O_{38}$	2635.85	2635.54	8.964[10]
R4	SDITVGQYGGNNAALVNQTASD	$C_{90}H_{144}N_{28}O_{36}$	2195.30	2195.50	8.350[11]
R5	SSVMVRQVGFGNNATANQY	$C_{86}H_{136}N_{28}O_{28}S$	2043.26	2043.72	7.054[12]
R1	SELNIYQYGGGNSALALQTDARN	$C_{104}H_{164}N_{32}O_{37}$	2455.64	2455.94	7.763[4]
R2	SDLTITQHGGGNGADVGQGSDD	$C_{82}H_{130}N_{28}O_{37}$	2101.10	2101.38	9.600[5]
R3	SSIDLTQRGFGNSATLDQWNGKN	$C_{106}H_{166}N_{34}O_{37}$	2509.70	2509.81	11.772[6]
R4	SEMTVKQFGGGNGAAVDQTASN	$C_{88}H_{142}N_{28}O_{34}$	2169.33	2169.37	8.350[5]
R5	SSVNVTQVGFGNNATAHQY	$C_{85}H_{129}N_{27}O_{29}$	1994.12	1994.46	6.861[7]

Blue color corresponds to *S. enterica*, black to *E.coli*.

[1] $M_{t.}$ – theoretical mass of peptide

[2] M_{MS} – found mass of the peptide using MALDI-TOF

[3] HPLC $t_{ret.}$ – retention time in analytical HPLC spectra (C18 column 250x4.6 mm, detection wavelength 220 nm, buffer A: 0.05% TFA + 2% CH_3CN; buffer B: 0.05% TFA + 90% CH_3CN)

[4] gradient 32-45% B in 13 min

[5] gradient 12-28% B in 16 min

[6] gradient 8-26% B in 18 min

[7] gradient 20-38% B in 18 min

[8] gradient 22-38% B in 16 min

[9] gradient 18-29% B in 11 min

[10] gradient 22-36% B in 14 min

[11] gradient 20-34% B in 14 min

Table S6. Peptides analytical data synthesized "in house".

Name	Sequence	Formula	$M_{t.}$[1]	M_{MS}[2]	HPLC $t_{ret.}$[3] [min]
R2	SDLTITQHGGGNGADVGQGSDD	$C_{82}H_{130}N_{28}O_{37}$	[1/2M+1] 1050.97 [1/3M+1] 700.98	[1/2M+1] 1051.47 [1/3M+1] 701.59	11.738
R5	SSVNVTQVGFGNNATAHQY	$C_{85}H_{129}N_{27}O_{29}$	[1/2M+1] 997.48 [1/3M+1] 665.32	[1/2M+1] 997.98 [1/3M+1] 665.98	12.004

[1] $M_{t.}$ – theoretical mass of peptide

[2] M_{MS} – found mass of the peptide using ESI

[3] HPLC $t_{ret.}$ – retention time in analytical HPLC spectra (column ReproSil Saphir C18 100A 5µ 150 x 4.6 mm; detection wavelength 222 nm; A = H_2O, B = CH_3CN, gradient: t=0–20 min, 90%–0% A; t=20–25 min, 0% A; t=25–30 min, 0%–90%

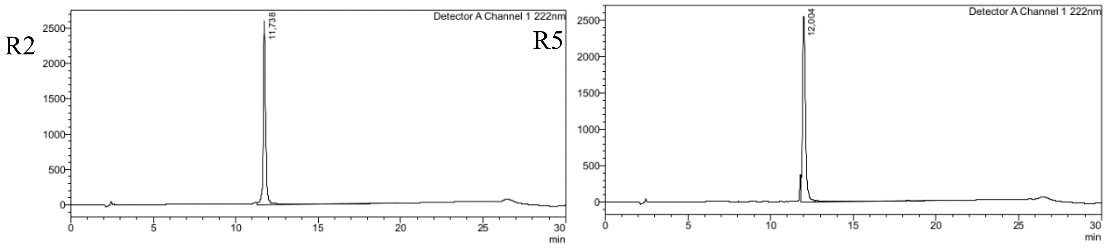

Figure S4. Analytical HPLC chromatograms of "in house" studied peptide

Table S7: Raw ATR-FTIR spectra of *E.coli* fragments in the range of 3600-900 cm^{-1} .

<table>
<tr><th colspan="2">E. coli</th></tr>
<tr><th>0</th><th>30</th></tr>
<tr><td>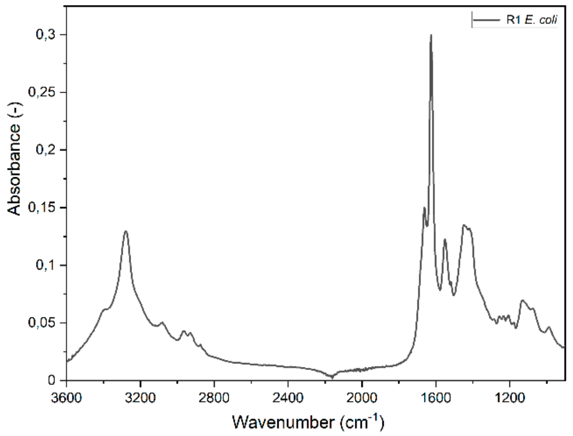
</td><td>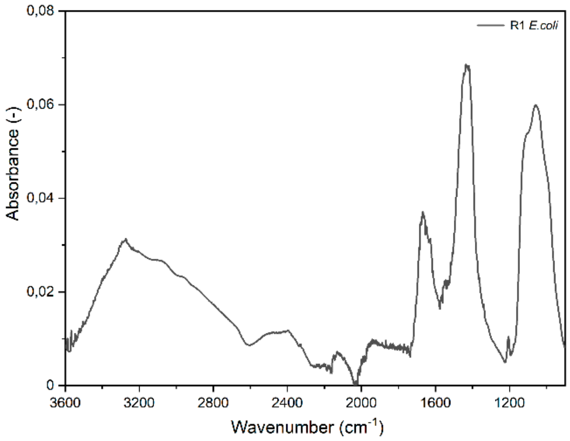
</td></tr>
<tr><td>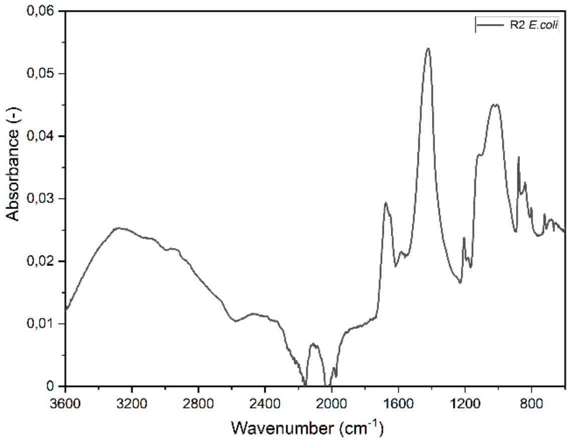
</td><td>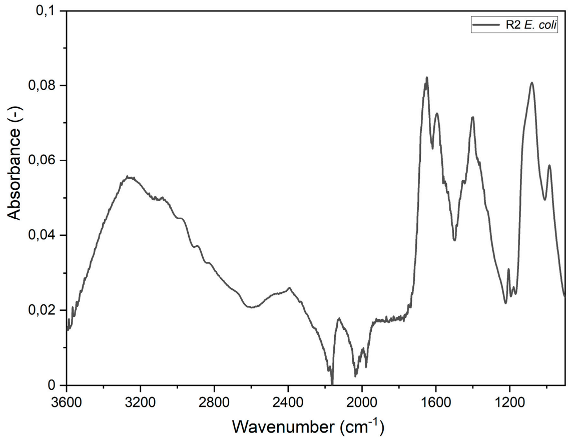
</td></tr>
<tr><td>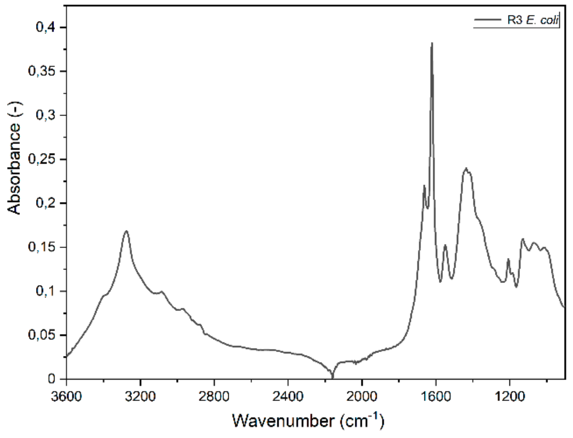
</td><td>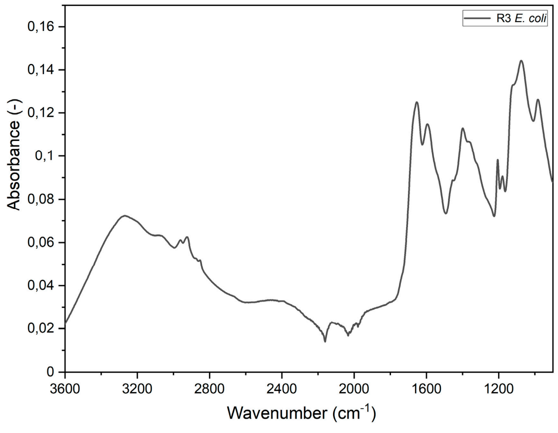
</td></tr>
</table>

<table>
<tr><td>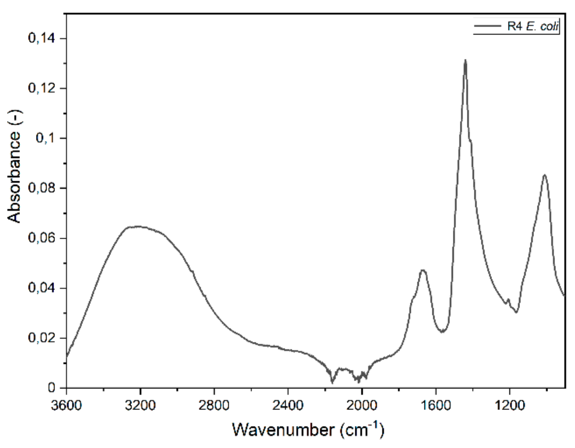
</td><td>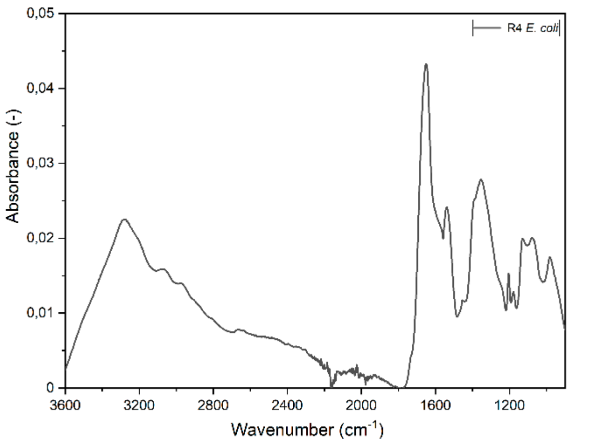
</td></tr>
<tr><td>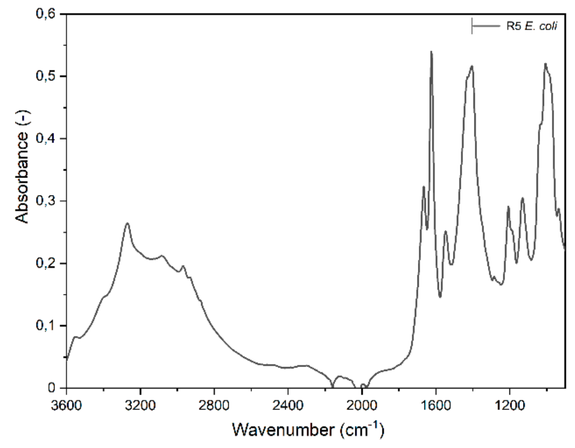
</td><td>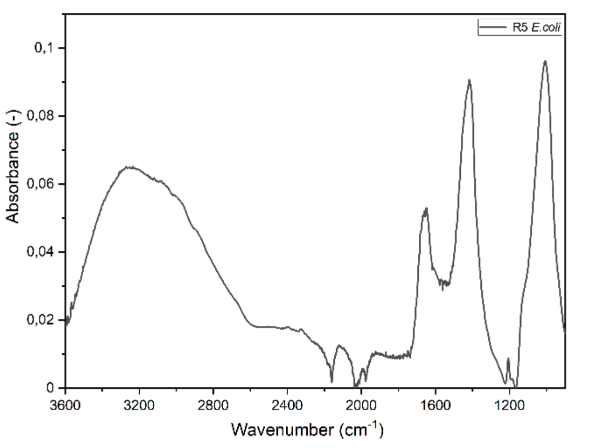
</td></tr>
</table>

Table S8. Raw ATR-FTIR spectra of *S. enterica* fragments in the range of 3600-900 cm^{-1} .

<table>
<tr><th colspan="2">S. enterica</th></tr>
<tr><th>0</th><th>30</th></tr>
<tr><td>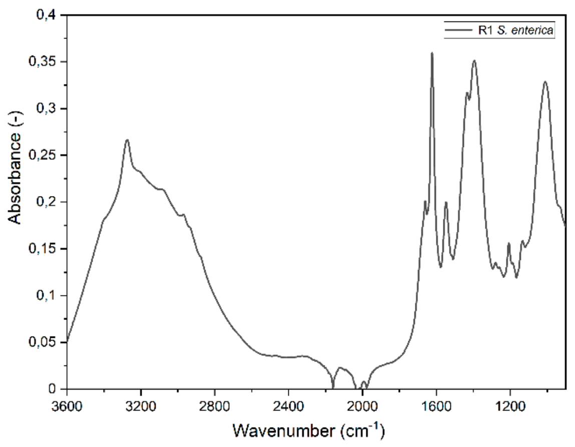
</td><td>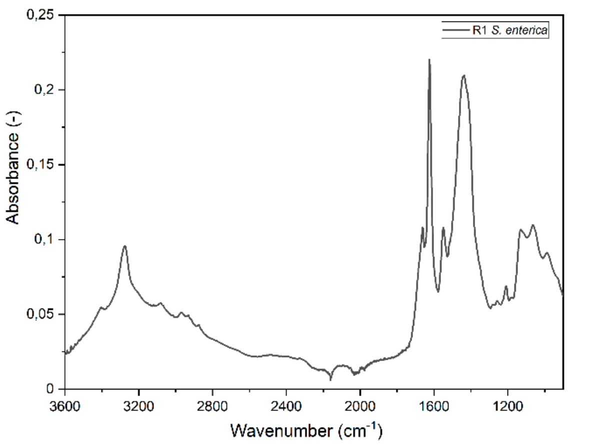
</td></tr>
</table>

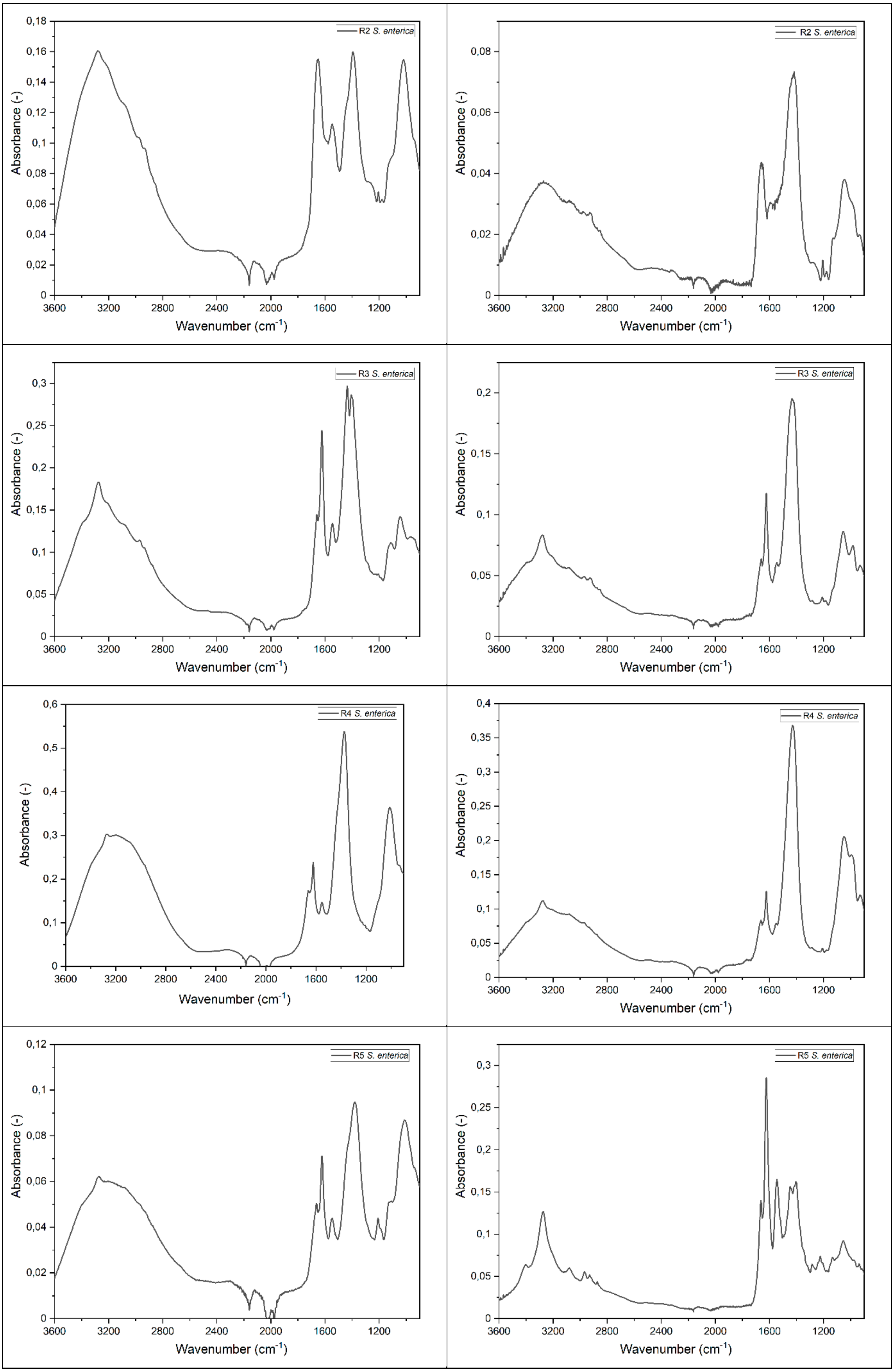
R2 S. enterica
Absorbance (-)
Wavenumber (cm-1)
R2 S. enterica
R3 S. enterica
R3 S. enterica
R4 S. enterica
R4 S. enterica
R5 S. enterica
R5 S. enterica

Table S9. Raw FT-Raman spectra of *E.coli* fragments in the range of 3600-600 cm^{-1} .

<table>
<tr><th colspan="2">30</th></tr>
<tr><th>E. coli</th><th>S. enterica</th></tr>
<tr><td>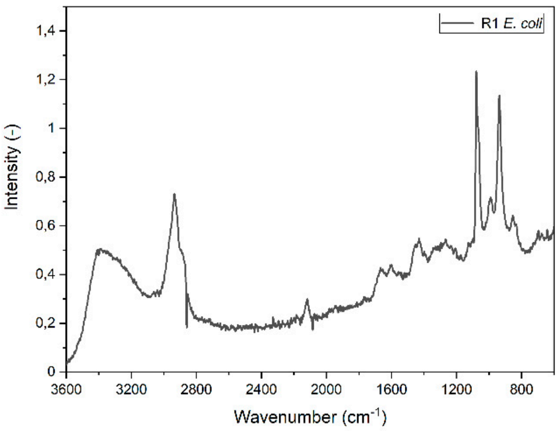
</td><td>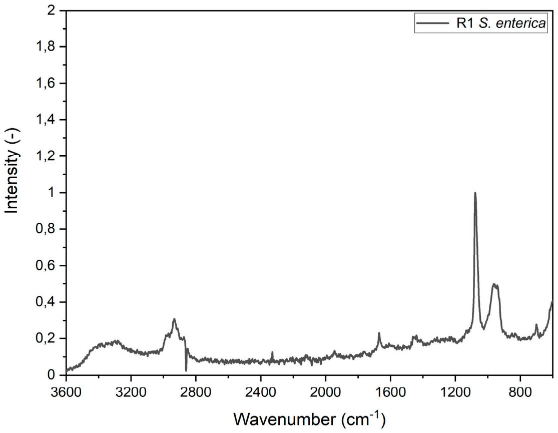
</td></tr>
<tr><td>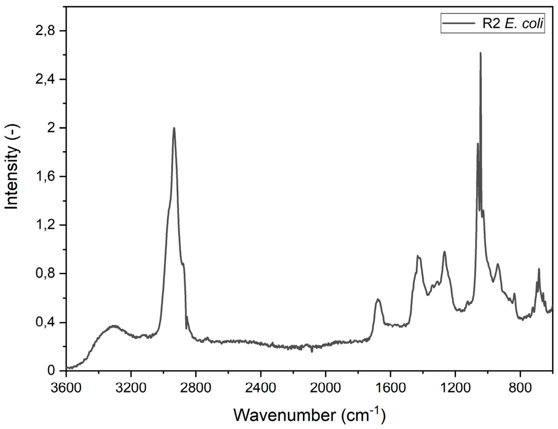
</td><td>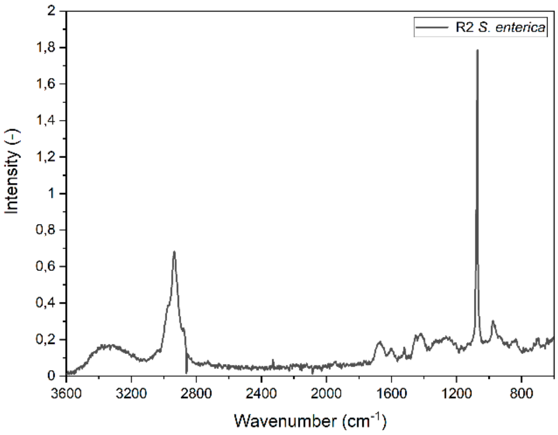
</td></tr>
<tr><td>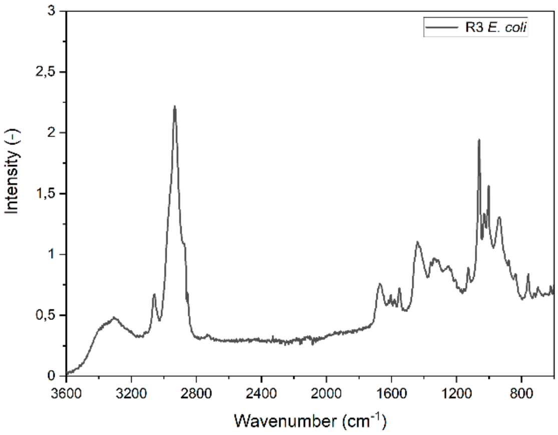
</td><td>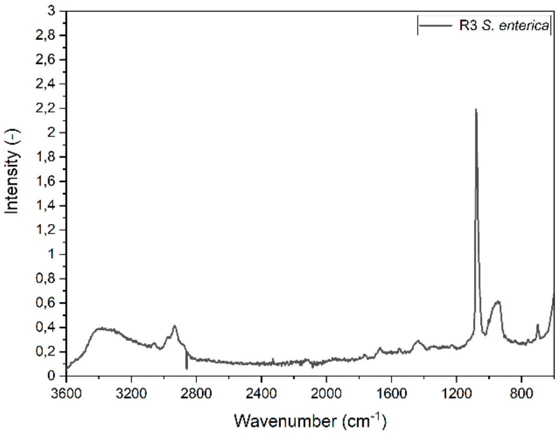
</td></tr>
</table>

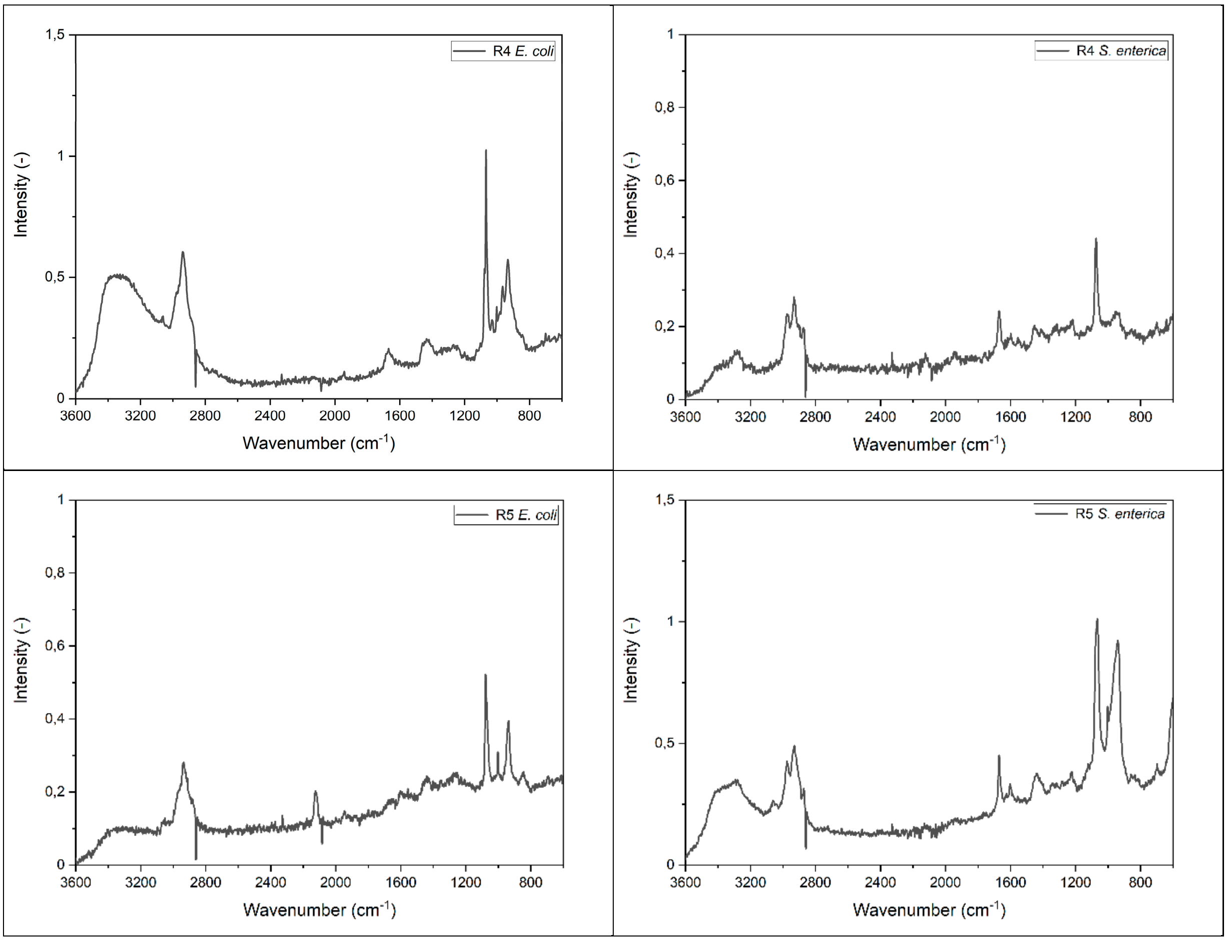

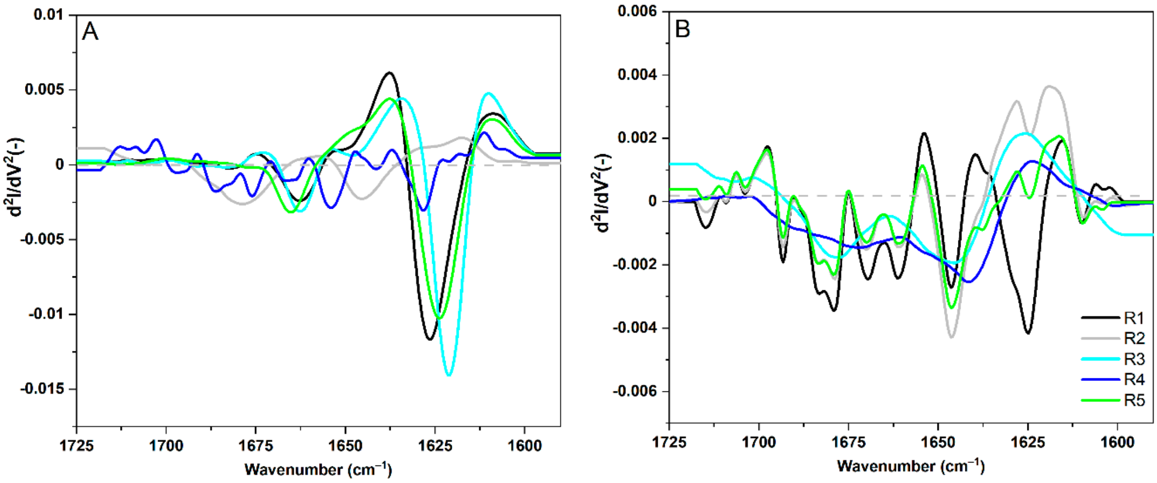

Figure S5. ATR-FTIR second derivatives spectra of *E. coli* fragments, in the wavenumber range of 1725-1590 cm^{-1}, smoothed twice with SG 35 (see Methods). (A) on the day of the dissolving (B) after month incubation at 37 °C. Peptide concentration was 500 μM.

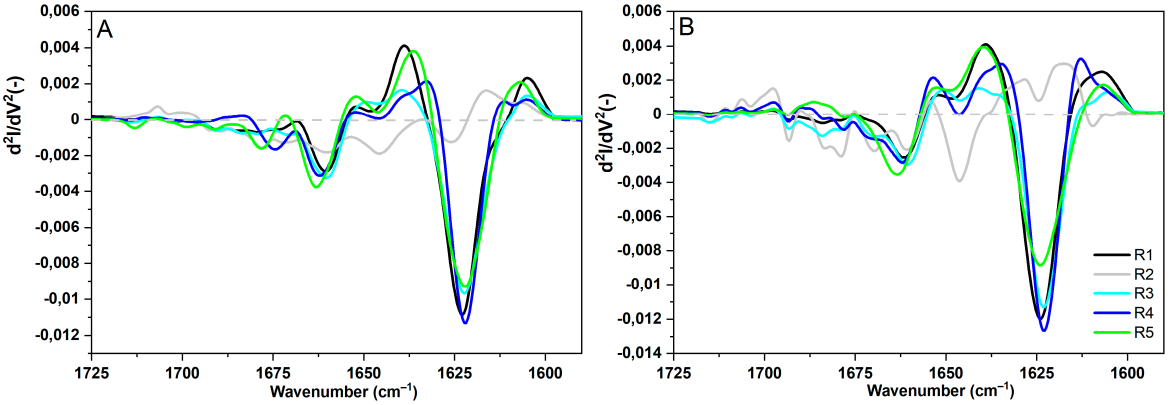

Figure S6. ATR-FTIR Second derivatives spectra of *S. enterica* fragments, in the wavenumber range of 1725-1590 cm^{-1}, smoothed twice with SG 35 (see Methods). (A) on the day of the dissolving (B) after month incubation at 37 °C. Peptide concentration was 500 μM.

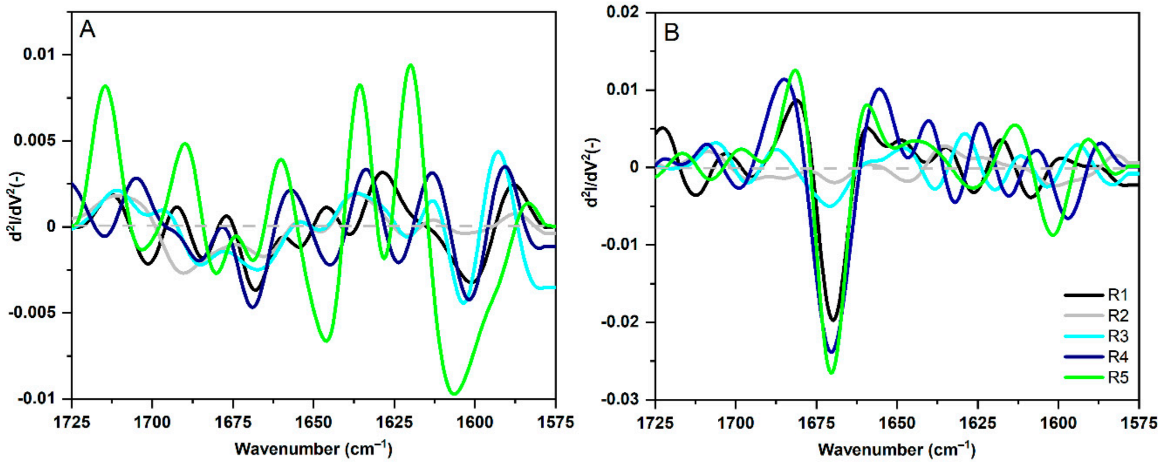

Figure S7. FT-Raman second derivatives spectra, smoothed twice with SG 35 (see Methods), in the wavenumber range of 1725-1575 cm^{-1}. (A) Spectra for *E. coli* fragments after 30 days of incubation at 37 °C , (B) Spectra for *S. enterica* fragments after 30 days of incubation at 37 °C. Peptide concentration was 500 μM.
